# Probing Loop-Mediated Isothermal Amplification (LAMP) targeting two gene-fragments of rose rosette virus

**DOI:** 10.1371/journal.pone.0256510

**Published:** 2021-11-29

**Authors:** Andrea Salazar, Francisco M. Ochoa-Corona, Jennifer D. Olson, Binoy Babu, Mathews Paret

**Affiliations:** 1 Oklahoma State University, Stillwater, Oklahoma, United States of America; 2 Institute for Biosecurity and Microbial Forensic, Stillwater, Oklahoma, United States of America; 3 Department of Entomology and Plant Pathology, Stillwater, Oklahoma, United States of America; 4 Universidad de las Fuerzas Armadas- ESPE, Quito, Ecuador; 5 North Florida Research and Education Center, University of Florida, Quincy, Florida, United States America; Bangladesh Agricultural University, BANGLADESH

## Abstract

This study explores the development of Loop-mediated isothermal amplification (LAMP) for detection of rose rosette virus (RRV), a technique with the potential to be translated to rose nurseries. RRV is a negative-sense, single-stranded RNA virus which is a member of the genus *Emaravirus* (Family *Fimoviridae*) and the causal agent of the rose rosette disease (RRD). Although RRV symptoms are characteristics, early visual diagnosis of RRD can be misleading and confusing since it may appear like herbicide damage. Moreover, it may take incubation time for symptoms to appear after virus infection. Two sets of RRV gene sequences RNA3 and RNA4 were analyzed and two sets of four LAMP primers were designed. The direct antigen-capture method for direct trapping of RRV in plastic was used for RNA extraction followed by cDNA synthesis. RT-LAMP reactions were for 1 hour at 64°C (RRV-P3) and 66.5°C (RRV-P4) using either a thermocycler or a portable dry bath. RT-qLAMP was also optimized using DNA polymerase GspSSD LD using the same RRV sets of primers. RRV was detected in symptomatic and non-symptomatic RRD tissue from Oklahoma. The limit of detection (LoD) was 1pg/μL and 1 fg/μL using Bst 2.0 LAMP and GspSSD LD quantitative LAMP, respectively. In visual colorimetric pre- and post-reactions, the LoD was 10 pg/μL and 0.1 pg/μL using hydroxy naphthol blue (HNB, 120 μM) and SYBR green I (1:10 dilution), respectively. No cross-reactivity was detected in the RT-LAMP reaction testing cDNAs of eight commonly co-infecting rose viruses and one virus taxonomically related to RRV. Four different dyes were tested, and visible colorimetric reactions were obtained with RT-LAMP Bst 2.0 combined with SYBR I or HNB. RT-qLAMP with GspSSD2.0 offers LoD equal to RT-PCR and it is faster since it works with RNA directly.

## 1. Introduction

Roses are affected by a wide variety of fungi, bacteria, viruses, nematodes, and phytoplasmas, which cause growth reduction, defoliation, and plant death [1]. In most cases, these pathogens attack flowers or leaf tissue causing economic losses to landscapers, gardeners, public gardens, and nurseries [1]. In the United States, the total of shrub rose wholesale was 28 million [2]. Rose rosette disease (RRD) is one of the most devastating diseases of roses. This disease was reported in 1940 in Manitoba, Canada, and it has since spread to roses in Kansas, Oklahoma, Missouri, and Arkansas [3]. Currently, RRD is endemic in the north-central, south-central, and southeastern regions of the U.S. [4, 5]. In 2012, thousands of roses at the Tulsa municipal rose garden expressed characteristic symptoms of the RRD and were eliminated after the damage inflicted by the disease; also as a precautionary sanitary practice to maintain the beauty of the garden [6]. Since then, the rose garden never recovered, all roses got lost and the garden lots have been used to assess rose varietal resistance to RRD.

Rose rosette virus (RRV) is a negative-sense, single-strand RNA virus, member of the genus *Emaravirus*, (Family *Fimoviridae*) [4]. The eriophyid mite *Phyllocoptes fructiphilus* is the known vector that transmits RRV [7, 8]. RRV appeared in populations of wild multiflora roses in the U.S. *Rosa multiflora* is a susceptible host and reservoir for virus and vector [9]. This rose species is invasive and was introduced from Asia, mainly from Japan [9]. RRV has seven genomic RNA segments: RNA1 (RNA-dependent RNA polymerase), RNA2 (glycoprotein), RNA3 (nucleocapsid protein), RNA4 (movement protein), and RNA5, RNA6, and RNA7 specific functions are yet to be determined [4, 7, 10]. RRD symptoms may be obvious from mid-spring to fall when plants are foliated; symptom expression depends mainly on the rose cultivar, age of the plant, and growing conditions. The RRV diagnosis might be misleading at the early stages of the disease [11]. Symptoms of RRD are diverse and include red abnormal coloration of shoots and foliage, sprout elongation, excessive thorn proliferation, and witches’ broom [11]. In addition, internodes are short, leaves are dwarfed, elongated or misshapen, flowers may be mottled, or distorted, secondary branches are thicker compared to primary branches, chlorosis on leaves reduced resistance to winter [11]. Plant dead may occur in one or two years [11, 12], apparently, rose plants lose resistance to winter conditions.

The RRV detection by symptoms can be only performed if the plant is expressing the unique symptoms associated with RRD. However, the best detection methods for presumed multiple virus presence require performing molecular detection assays, bioassays, etc, which are more laborious to process if compared to diagnostic procedures in use for other phytopathogens. The selection of the detection methods applied to plants, or virus vectors, plays an important role in the final diagnostic output and management of viral diseases [13, 14]. One of the most utilized techniques is RT-PCR (Reverse Transcription-Polymerase Chain Reaction) which exponentially amplifies target-specific diagnostic sequences, due to the high specificity and low LoD [15]. However, this technique requires costly laboratory equipment and trained personnel. Alternatively, isothermal DNA amplification methods amplify the targeted diagnostic sequence at a constant temperature, which improves cost and time in the detection method keeping the LoD and specificity the same as PCR [16, 17]. The most commonly used isothermal methods are Loop-mediated isothermal amplification (LAMP) and recombinase polymerase amplification (RPA). The last allows reactions at a lower temperature (37°C) that can be either fluorometric or qualitative developed in a lateral flow device, however, it may be costly [18].

LAMP is an isothermal method that achieves a high degree of specificity and efficiency. LAMP uses a DNA polymerase and a set of four to six oligonucleotides specifically designed to recognize six to eight regions of the diagnostic target DNA, respectively [15]. In addition, LAMP results can be visualized by the naked eye using either a pre-reaction and pH-sensitive dye (Hydroxynaphthol Blue) or a post-reaction fluorescent dye (PicoGreen or SYBR Green I) [19, 20].

Among, the best management practices once the RRD occurs is to remove symptomatic plants including the root ball seeking to minimize secondary inoculum and the spreading of viruliferous mites to healthy plants, which in time causes economic losses [1]. Accurate, sensitive, and field transferable detection of RRV is needed for early detection of RRV in foundation blocks, susceptible breeding lines, and biosecurity purposes. This study hypothesizes the technological need can be fulfilled by exploring the development of a LAMP method while investigating chemistries and dyes available for naked eye visualization.

## 2. Material and methods

**Fig 1:**
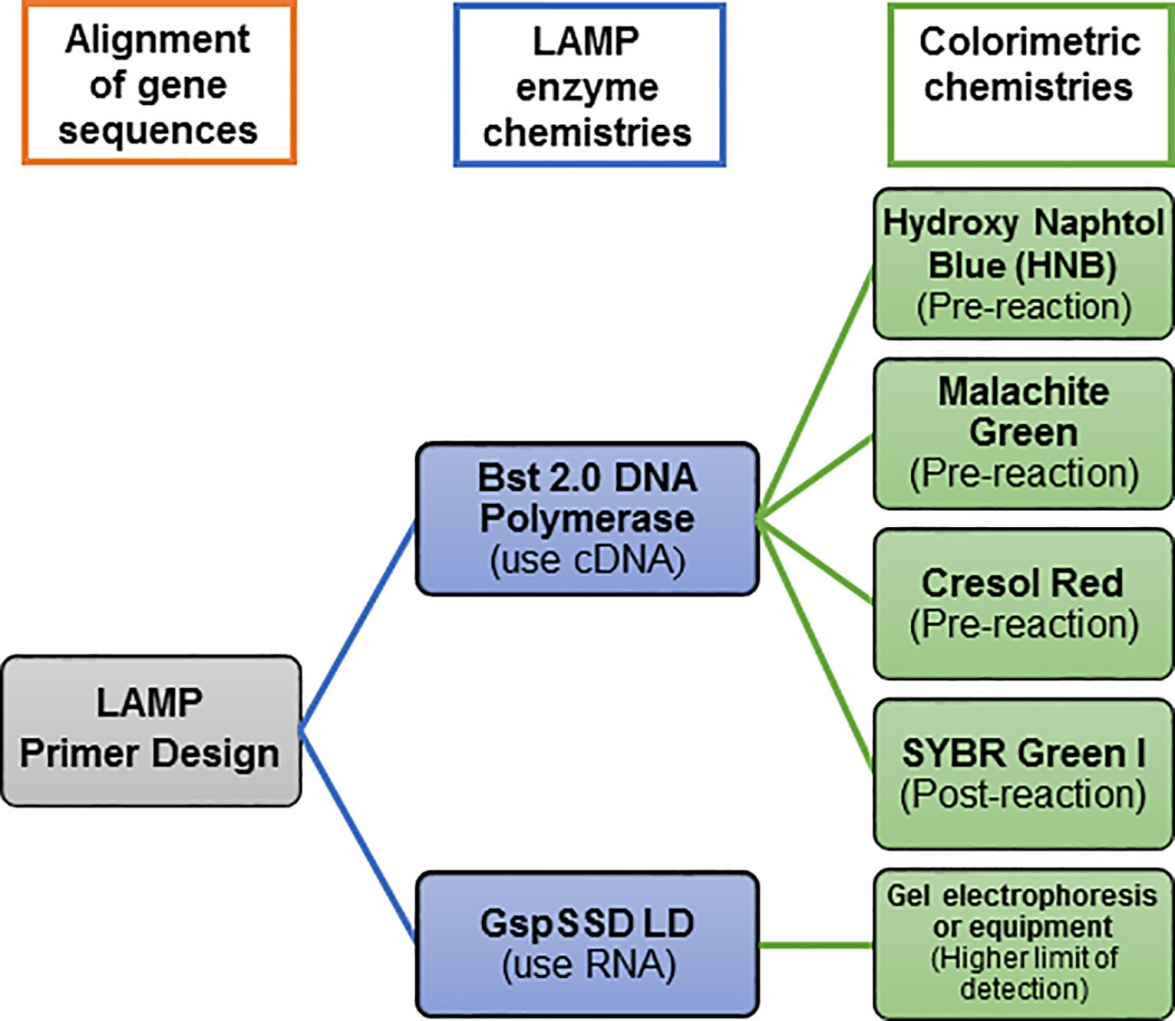
Flow chart showing the steps, methods, and chemistries used to explore Reverse Transcription Loop-Mediated Isothermal Amplification (RT-LAMP) targeting two genes of rose rosette virus (RRV) for detection of the virus.

A flow chart showing the RRV genes targeted, and the sequence of methods and chemistries explored is shown in Fig 1.

### 2.1. Source of viruses

Infected plant tissue was from Oklahoma where RRV is endemic. Samples were sourced by the Plant and Insect Disease Diagnostic Laboratory (PDIDL), Oklahoma State University (OSU), and were collected from two RRV resistance rose-varietal plots located in Perkins and the Tulsa rose garden, Oklahoma. Nine lyophilized reference-positive control of viruses commonly infecting roses were from Agdia, Inc (Agdia, Elkhart, IN) and used for specificity assays. These virus species are impatiens necrotic spot virus (INSV), High Plains wheat mosaic virus (formerly High Plain virus) (HPWMoV), arabis mosaic virus (ArMV), maize stripe virus (MSpV), tomato spotted wilt virus (TSWV), apple mosaic virus (ApMV), prunus necrotic ringspot virus (PNRSV), tomato ringspot virus (ToRSV), and tobacco mosaic virus (TMV). Healthy tissue of *Rosa multiflora* was used as a negative control.

### 2.2. LAMP primer design

Twenty-two RRV nucleoprotein gene sequences (RNA3) were retrieved from the NCBI GenBank. The accession numbers of the analyzed sequences are HQ891892.1- HQ891913.1. Twenty sequences of the RRV movement protein gene (RNA4) were retrieved from the NCBI GenBank database. The accession numbers of analyzed sequences are HQ891870.1—HQ891889.1. The last date of accession was November 17th, 2019.

The NCBI accessions selected for LAMP primers design were RRV isolates collected in Arkansas, Mississippi, Missouri, Alabama, Tennessee, Iowa, and Oklahoma.

Sequences of LAMP oligonucleotides primers were designed using the web interface application Primer Explorer (Eiken Chemical Co., Ltd.) (http://primerexplorer.jp/e/). A set of LAMP primers was selected (Table 1) following parameters described in the Primer Explorer Manual (S1 Table). The specificity of the LAMP primers was tested *in silico* using BLASTn [21]. LAMP primers were synthesized by Integrated DNA Technologies (Coralville, IA, USA).

**Table 1:** General thermodynamic characteristics of the LAMP primers designed for rose rosette virus (RRV) and calculated by primer explorer.

RRV gene	LAMP Primer	Primer Code	Sequence (5’ –3’)	Location (nt)	Tm (°C)	GC %	Self compl.	Self-3’ compl.
**P3 (RNA 3)**	**F3**	**RRVP3-F3**	AGAAGCCTTCGAAGATCG	1230–1253	54.11	50.00	8.00	3.00
	**B3**	**RRVP3-B3**	AATCTCTGAAGTAAAAGGTGTAG	1406–1423	53.80	34.78	3.00	0.00
	**FIP**	**RRVP3-FIP**	CGAAGCTTCTGATCAGCTCCGA-AAATCCTGGAACAAGCACA	1333–1354 1386–1403	67.3	48.8	*	*
	**BIP**	**RRVP3-BIP**	GGTCCTCAAGTTGACAAATGTTCA-GTTCAATATAAACTGGGTCCAATT	1309–1332 1253–1276	64.5	37.5	*	*
**P4 (RNA 4)**	**F3**	**RRVP4-F3**	ATTGTTGGCTCAGGGGAA	1061–1078	55.95	50.0	3.00	0.00
	**B3**	**RRVP4-B3**	ATCCAGCTGTAGATTGAGTT	885–904	53.73	40.0	6.00	0.00
	**FIP**	**RRVP4-FIP**	ACGAATTGTTGGAAATTTGGATCAA-GCTTAATCTTGATCTTATGGGAAC	992–1016 1037–1060	63.7	34.7	*	*
	**BIP**	**RRVP4-BIP**	CAGGCTCACTTGATTTTGCAACTG-CACCCATCCTAGTATCAGG	966–989 906–924	66.9	48.8	*	*

*The value of this parameter is not calculated by Primer Explorer.

### 2.3. RNA extraction

RNA extractions were made from both fresh or lyophilized rose tissue and commercially available virus reference controls (Agdia, Elkhart, IN). For fresh tissue, approximately 100 mg of leaves, petals, bark, or roots were loaded in 2 mL microcentrifuge tubes and pulverized using liquid nitrogen and mini-pestels. Total RNA was extracted using the RNeasy plant mini kit (Qiagen Inc., Valencia, CA) following the instructions of the manufacturer. For Agdia lyophilized reference positive controls, 450 μL of RLT buffer (from the Qiagen RNeasy plant mini kit) was directly aliquoted into the positive control vial, vortexed for 30s, and the total RNA extracted following the manufacturer’s instructions.

### 2.4. Virus direct antigen-capture method

Viral RNA was obtained from leaves, petals, bark from young stems, and roots using direct antigen-capture or direct trapping in plastic as described by Babu et al. [18]. Briefly, rose tissue was macerated in 1 mL of phosphate buffer saline (1X PBS) (VWR, Radnor, PA), 0.05% Tween 20, and pH 7.4. Fifty microliters of sap extract were aliquoted into a sterile PCR tube (0.5 ml polypropylene), avoiding air bubbles. The sap was incubated on ice for 2 min, then, the PCR tubes were rinsed twice with 50 μL of 1X PBS-T buffer. To release the viral RNA from the plastic-surface captured virion, 30 μL of nuclease-free water with RNAsin (Promega, Madison, WI) (100 U/mL, final concentration) was added and incubated at 95°C for 1 min. The supernatant was directly used in reverse transcription reactions.

### 2.5. cDNA synthesis

Four microliters of extracted total RNA obtained either with an RNA extraction kit or the direct antigen-capture method were used. The protocol was performed in two steps. First, denaturation followed by reverse transcription. LAMP RRVP3-B3 or RRVP4-B3 primers (5 μM) were used rather than random hexamer primers, and Moloney Murine Leukemia Virus Reverse Transcriptase (M-MLV RT) (Promega, Madison, WI), following the manufacturer’s instructions. cDNA synthesis was performed at 37°C for 90 min.

### 2.6. RT-PCR and cloning of RRV-P3 and RRV-P4 fragments

RT-PCR products were amplified with both LAMP external primers RRVF3- B3 or RRVF4- B4. The PCR reaction mix used was: 10 μL GoTaq Green Master Mix (Promega, Madison, WI), 1 μL RRVP3/F3 and RRVP3/B3 or RRVP4/F3 and RRVP4/B3 primers (5 μM), 3 μL of cDNA template, 1.6 μL of BSA (bovine serum albumin, 50 mg/mL; Ambion, Austin, TX), 2 μL 10% PVP40 (polyvinylpyrrolidone, Sigma-Aldrich, St. Louis, MO), and 1.4 μL nuclease-free water (Promega, Madison, WI). The final volume of the PCR reaction was 20 μL. The RT-PCR reaction was performed in a thermal cycler (Biometra, Goettingen, Germany) and cycling parameters were as follows: initial denaturation of 94°C for 5 min, 40 cycles of denaturation at 94°C for 20 s, annealing at 56°C for 20 s, extension at 72°C for 20 s, and a final extension at 72°C for 5 min. The amplified products were analyzed by 1X TAE-2% agarose gel electrophoresis (Tris-acetate-EDTA) and stained with SYBR Safe (Invitrogen, Waltham, MA) according to the manufacturer. The 100bp DNA Ladder (Promega, Madison, WI) was added in all the gel electrophoresis analyses.

The RRV-P3 and RRV-P4 RT-PCR products were excised and purified from the agarose gel in two stages. First, by QuantumPrep Freeze’N Squeeze Spin Columns (Bio-Rad, Hercules, CA), followed by a second purification with High Pure PCR Product Purification Kit (Roche, Germany). The TOPO TA cloning kit (Invitrogen, Waltham, MA) was used for cloning the purified PCR fragments segments of RRV-P3 and RRV-P4 genes. The TOPO TA kit was used according to the manufacturer’s instructions. Briefly, the previous purified PCR products were ligated into the commercial pCR´4-TOPO plasmid and transformed into *Escherichia coli* cells (TOP10, Mach1™-T1R, DH5α™-T1R cells) following the manufacturer’s instructions. Plasmids were sequenced by Sanger sequencing and stored at -20°C until use.

### 2.7. RT-LAMP optimization

The purified plasmids carrying the RT-PCR inserts of RRV-P3 and RRV-P4 were used for optimization purposes. The optimal temperature was assessed from a gradient of temperature assay from 60°C to 72°C and the optimal MgSO4 concentration was determined by testing nine different concentrations from 2mM to 10mM.

RT-LAMP reagents were: 2.5 μL isothermal amplification buffer (1X; Biolabs, Ipswich, MA), 2.5 μL betaine (0.8 M; Lucigen, Middleton, WI), 1 μL MgSO4 (4 mM; Biolabs, Ipswich, MA), 1.4 μL dNTPs (1.4 mM, GeneScript, Piscataway, NJ), 1 μL LAMP primers F3 –B3 (0.2 μM), 1 μL AMP primers FIB–BIP (0.8 μM), 1 μL Bst 2.0 WarmStart^®^ DNA polymerase (0.32 u; Biolabs, Ipswich, MA), 1 μL Hydroxy Naphthol Blue (HNB) (120 μM; Sigma- Aldrich, St. Louis, MO), 1 μL RRV-P3/P4 plasmid, 3 μL of cDNA from rose tissue and 3 μL nuclease-free water (Promega, Madison, WI) were used as the template for positive control, samples, and non-template control, respectively. The final volume of the LAMP reaction was 25 μL.

RT-LAMP was performed in a dry bath incubator (GeneMate/Bioexpress, Kaysville, UT). RT-LAMP reactions for RRV-P3 were incubated for 1 h at 64°C and 1h at 66.5°C for RRV-P4. The polymerase deactivation was performed at 80°C for 10 min, at the end of the reaction. The amplified products were analyzed by electrophoresis in 2% agarose gel, 1X TAE buffer, and stained with SYBR Safe (Invitrogen, Waltham, MA). In order to visualize the RT-LAMP products, two colorimetric reactions were studied and optimized. The pre-colorimetric reaction was assessed using HNB, which was included in the RT-LAMP mix reaction. On the other hand, the post-colorimetric reaction was performed using 3 μL of freshly prepared 10-fold dilution of SYBR Green I dye (Invitrogen, Waltham, MA), which was added after the RT-LAMP incubation period (Fig 1). Cresol red (16 mM) and Malachite green (0.2%) (Sigma-Aldrich, St Louis, MO) were also tested to assess their pre-colorimetric reaction using Bst 2.0 WarmStart^®^ DNA polymerase.

### 2.8. RT-quantitative LAMP

RT-qLAMP reactions were carried out in 15 μL isothermal GspSSD2.0 master mix (ISO-004) (OptiGene, Horsham, UK), 1 μL LAMP primers F3 –B3 (0.2 μM), 1 μL LAMP primers FIB–BIP (0.8 μM), and 1 μL RRV-P3 plasmid or 3 μL of RNA extracted from rose tissue. Nuclease-free water was used for non-template control (Promega, Madison, WI). The final reaction volume was 25 μL. The reaction was performed in a Rotor-Gene 6000 thermocycler (Corbett Research, Qiagen Inc., Valencia, CA). Rotor gene was set up to read RT-qLAMP amplicons every 1 minute for 50 min at 64°C (green channel). Enzyme deactivation was performed at 80°C for 10 min at the end of the reaction. The reaction data were registered and analyzed using the Rotor-Gene 6000 series and software 1.7 (Corbett Research, Qiagen Inc., Valencia, CA).

### 2.9. Limit of detection and specificity assays

LoD assays were performed using ten-fold serial dilutions from 1 ng/μL to 1 fg/μL of plasmids RRV-P3 and RRV-P4. The plasmid concentration was quantified using a Nanodrop 2000 (Thermo Scientific, Waltham, MA). One microliter of each dilution was used as a template for RT-LAMP.

For specificity, the LAMP primers were RNA extracted from nine lyophilized reference virus controls (Agdia, Elkhart, IN) listed above in the section ‘source of viruses’. Plasmid RRV-P3 (positive control), cDNA and RNA from healthy rose tissue (negative control), and nuclease-free water (non-template control) were included in both LoDs and specificity assays. The results of these assays were analyzed in 2% agarose electrophoresis in 1X TAE buffer, colorimetric reaction, and RT-qLAMP.

### 2.10. Screening of field samples

RRV-P4 RT-LAMP was tested with 38 field samples including symptomatic and non-symptomatic rose samples collected at the Oklahoma State University RRV resistance rose varietal plot by PDIDL. RRV-P3 RT-LAMP was tested with 30 samples of rose tissue collected at the Tulsa rose garden. The RT-LAMP reaction conditions were according to the optimized parameters tested and described above. Endpoint RT-PCR was performed as a reference and confirmatory assay using the RRV primers and cycling conditions described by Dobhal et al. [11].

## 3. Results

### 3.1. LAMP primer design

The primers were selected after analysis of the thermodynamic parameters described by the Primer Explorer manufacturer for the selection of LAMP primers (S1 Table). LAMP primer sets for RRV-P3 and RRV-P4 aligned with the consensus regions of twenty-two RRV-P3 sequences and twenty RRV-P4 sequences (Table 1). The result showed 100% identity with 100% query coverage for both groups of gene sequences of RRV (P3 and P4), after the alignment analysis of outer LAMP primers RRV-P3/F3, RRV-P3/B3, RRV-P4/F3, and RRV-P4/B3. Matches with other emaraviruses and virus species were not detected.

### 3.2. RT-LAMP optimization

The RT-LAMP optimization (primer concentration, temperature gradient, and MgSO_4_ concentration) was carried out with plasmid harboring fragments of RRV-P3 and RRV-P4 genes amplified by endpoint RT-PCR with outer LAMP primers RRVP3-F3, RRVP3-B3, RRVP4-F3, and RRVP4-B3. The sequencing output of the two cloned plasmids had 99% identity to the RNA3 and RNA4 of RRV isolates in the NCBI database.

#### 3.2.1. Primer concentration

**Fig 2:**
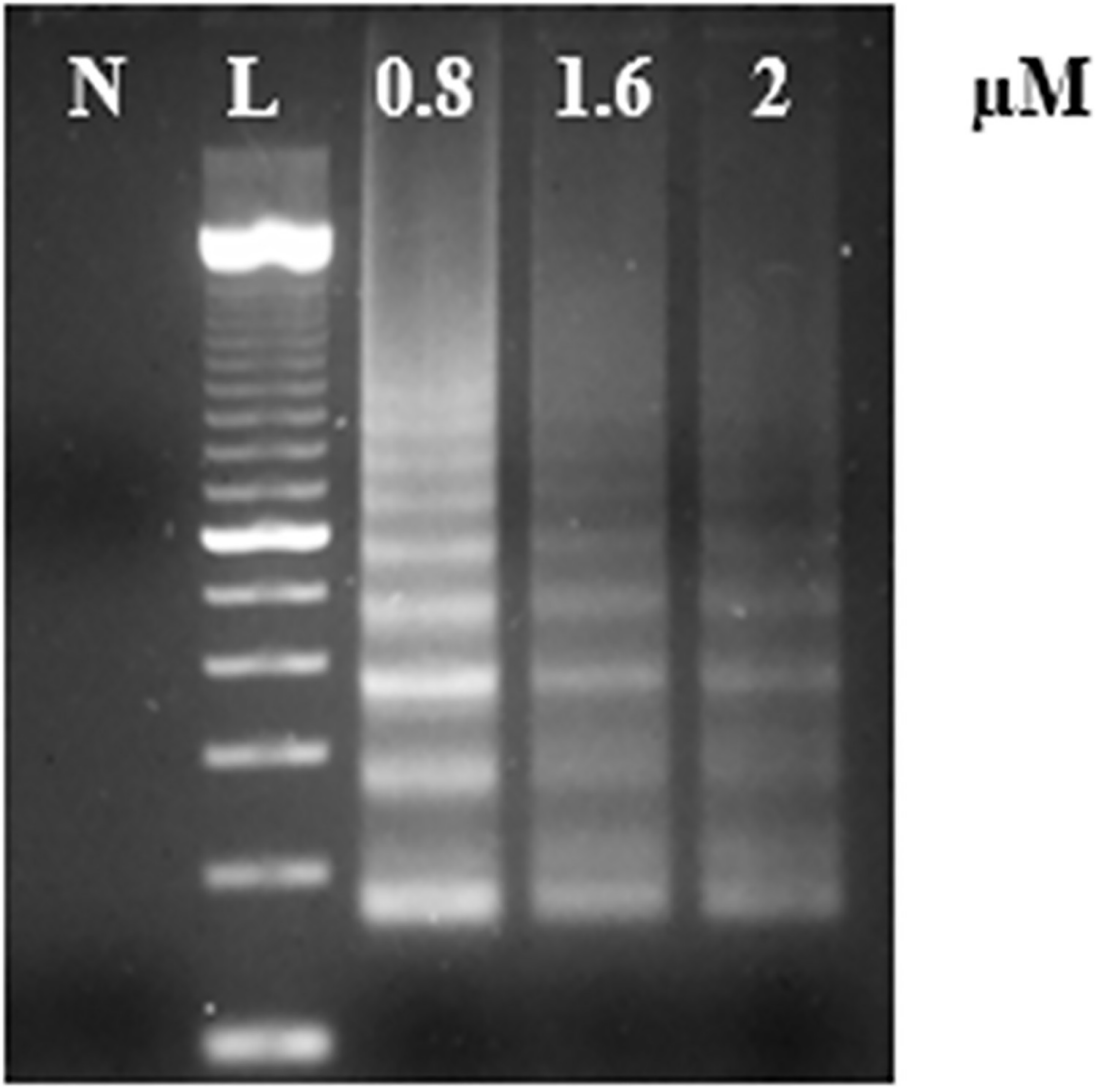
Optimization of the primer concentration using the outer LAMP primers RRVP3-FIP and RRVP3-BIP, the RRV-P3 plasmid as a template, and Bst 2.0 DNA Polymerase. Lane N, is a non-template control (NTC, water); lane L, is a 100 bp DNA ladder.

Since LAMP primer sets perform at different concentrations, three concentrations of the inner LAMP primers RRV-FIP and RRV-BIP (0.8, 1.6, and 2 μM) were tested with a single concentration of the outer primers RRV-F3 and RRV-B3 (0.2 μM). The best concentration of outer primers was 0.2 μM and 0.8 μM for inner primers, which produced the best visible and distinct band pattern indicative of a positive LAMP reaction amplification (Fig 2). We optimized the concentration for both sets of primers (RRV-P4 and RRV-P3) and the results were consistently the same for the two sets. Therefore, we only showed in Fig 1. the RRV-P3 results.

#### 3.2.2. RT-LAMP performance in temperature gradient

**Fig 3:**
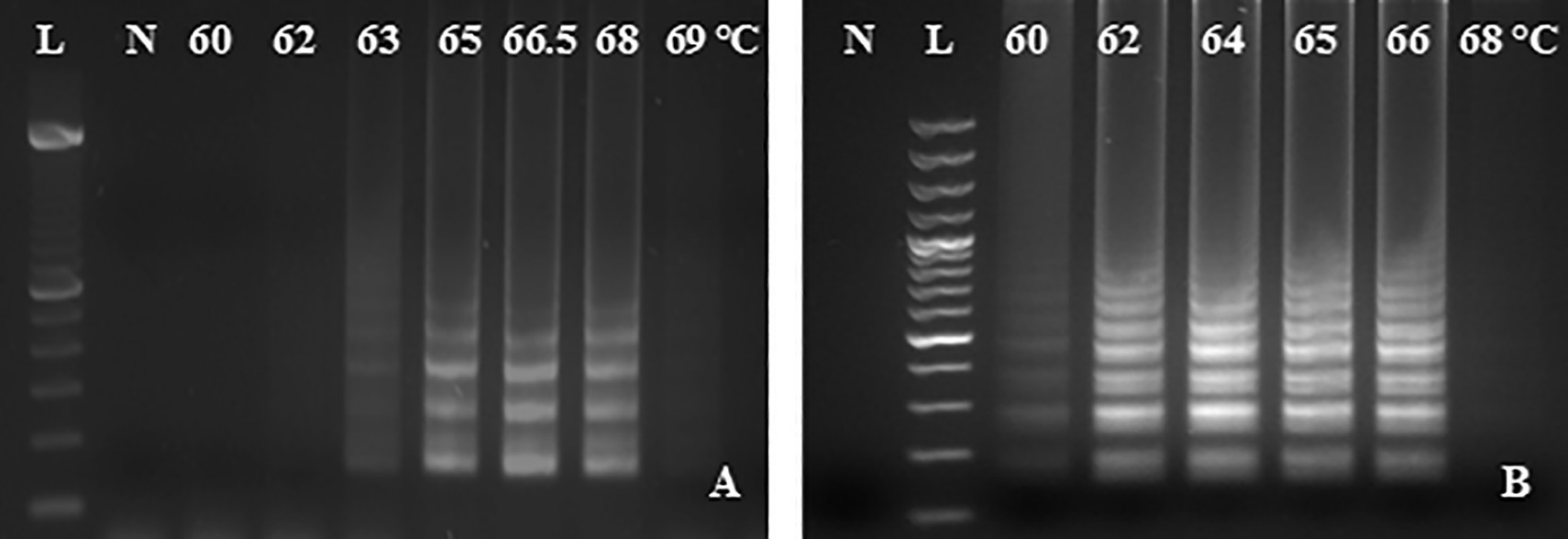
RT-LAMP temperature gradient assay from 60 to 69°C using Bst 2.0 DNA polymerase. A) RRV-P3 primers and RRV-P3 plasmid as template B) RRV-P4 primers and RRV-P4 plasmid as template. Lane L is a 100 bp DNA ladder and lane N, is a non-template control (NTC, water).

Primer set RRV-P3 amplified the expected product within 63 to 68°C (Fig 3A). Similarly, LAMP performed well in a broad range of temperatures amplifying the expected RRV-P4 gene diagnostic product from 60 to 66°C (Fig 3B). The annealing temperature ranged from five and six degrees Celsius respectively. The amplification temperatures selected for LAMP reactions were 64°C for RRV-P3 and 66.5°C for RRV-P4 for one hour. No product amplification was detected with the non-template control using the two LAMP primer sets.

#### 3.2.3. Concentration of magnesium sulfate

**Fig 4:**
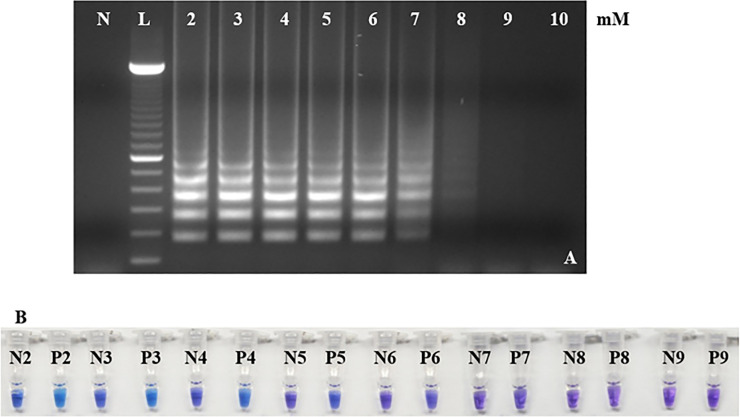
Effect of different concentrations of MgSO_4_ in RRV-P3 RT-LAMP using Bst 2.0 DNA polymerase. RRV-P3 and RRV-P4 RT-LAMP results are equal. (A) Gel electrophoresis of RRV-P3 RT-LAMP products. Lane 2 is 2 mM, 3 is mM, 4 is mM, 5 is 5mM, 6 is 6mM, 7 is 7 mM, 8 is 8 mM and 10 is 10 mM. Lane N, is a non-template control (NTC, water); lane L, is a 100 bp DNA ladder. The effect of MgSO_4_ concentrations in RRV-P4 LAMP was equal. (B) P tubes are RRV-P3 plasmid (1 ng/μL). P2 to P9 are colorimetric HNB reactions. Tube P2 is 2 mM, P3 is 3mM, P4 is 4 mM, P5 is 5 mM, P6 is 6 mM, P7 is 7 mM, P8 is 8 mM, and P9 is 9 mM. N tubes are non-template controls (NTC, water). Tube N2 is 2 mM, N3 is 3mM, N4 is 4 mM, N5 is 5 mM, N6 is 6 mM, N7 is 7 mM, N8 is 8 mM, and N9 is 9 mM.

The RRV-P3 and RRV-P4 diagnostic products were amplified within six MgSO4 concentrations: 2, 3, 4, 5, 6, and 7 mM (Fig 4A) using Bst 2.0 DNA polymerase and the pre-reaction and pH-sensitive dye HNB. The 4 mM MgSO4 concentration allowed the amplification and visual discrimination of RRV targets compared with non-template reactions. The same MgSO_4_ optimization results were obtained using both RRV-P3 and RRV-P4. The MgSO_4_ RRV-P3 RT-LAMP optimization is show in Fig 4. Negative reactions were purple and positive reactions were light blue (Fig 4B). MgSO_4_ concentrations 6–9 mM are not optimal (Fig 4A) and cannot be colorimetrically distinguishable (Fig 4B). Cresol red and Malachite green did not react in the colorimetric reaction using Bst 2.0 WarmStart DNA polymerase and no change of color was observed (data not shown).

### 3.3. RT-LAMP limit of detection

**Fig 5:**
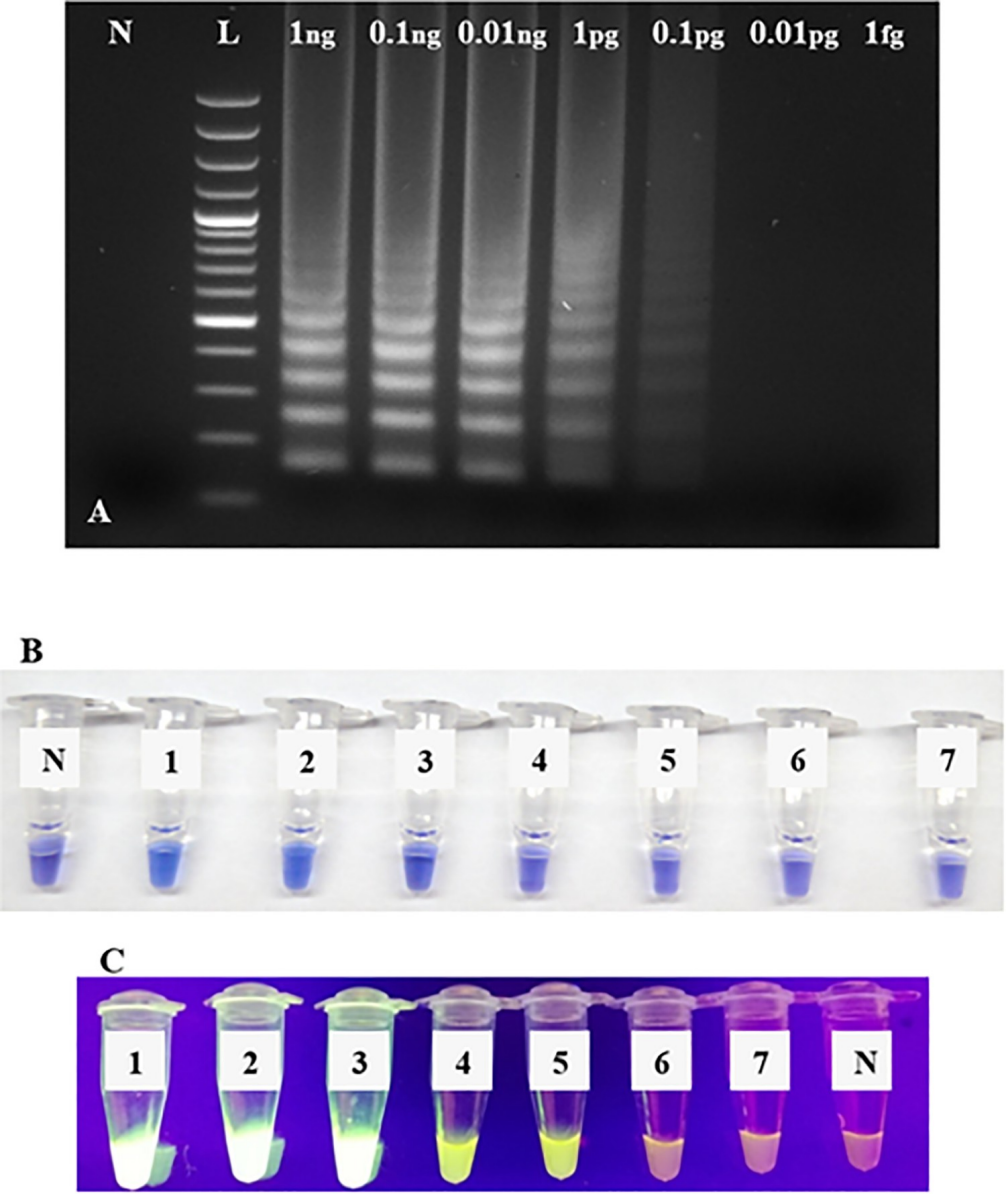
RRV-P3 RT-LAMP Limit of Detection (LoD) assays using Bst 2.0 DNA polymerase. (A) RT-LAMP LoD by gel electrophoresis using a ten-fold serially diluted RRVP-3 plasmid starting from 1ng/μL to 1fg/μL. Lane N, is a non-template control (NTC, water); lane L, is a 100 bp DNA ladder. (B) RT-LAMP colorimetric LoD using HNB and ten-fold serially diluted RRV-P3 plasmid starting from 1ng to 1fg. Tube N, is a non-template control (NTC, water); tube 1, 1ng/μL; tube 2, 0.1ng/μL; tube 3, 0.01ng/μL; tube 4, 1pg/μL; tube 5, 0.1pg/μL; tube 6, 0.01pg/μL; tube 7, 1fg/μL. (C) RT-LAMP colorimetric LoD using SYBR Green I and ten-fold serially diluted RRV-P3 plasmid starting from 1ng to 1fg. Tube 1, 1ng/μL; tube 2, 0.1ng/μL; tube 3, 0.01ng/μL; tube 4, 1pg/μL; tube 5, 0.1pg/μL; tube 6, 0.01pg/μL; tube 7, 1fg/μL. Tube N, is a non-template control (NTC, water).

**Fig 6:**
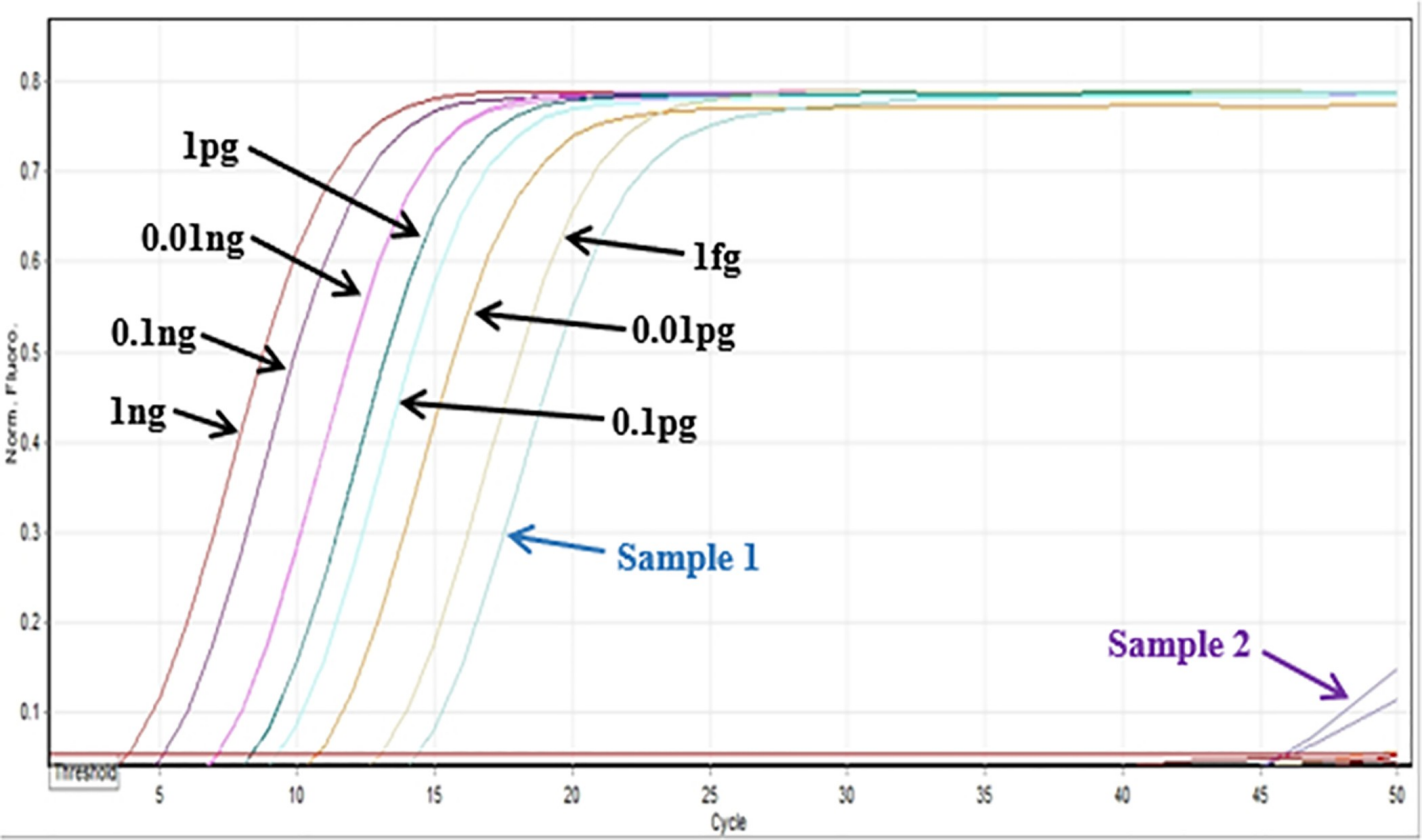
Limit of detection assays RT-qLAMP using GspSSD2.0 DNA polymerase. Limit of detection was made with a ten-fold serially diluted RRV-P3 plasmid starting from 1ng to 1fg and quantification of two unknown RNAs from rose tissue were tested (Sample 1 tested positive).

The concentration of the serial dilutions of plasmid RRV-P3 and RRV-P4were from 1 ng/μL to 1 fg/μL. The LoD of RRV-P3 using Bst 2.0 DNA Polymerase was 0.1 pg/μL after gel electrophoresis (Fig 5A). The colorimetric LoD using HNB was 10 pg/μL (Fig 5B) and 0.1 pg/ μL with SYBR green I (Fig 5C). The LoD of RT-qLAMP using GspSSD2.0 DNA polymerase was 1 fg/μL (Fig 6). A field sample was included in the assay and the RRV concentration was quantified (189 fg/μL) (Fig 6).

**Fig 7:**
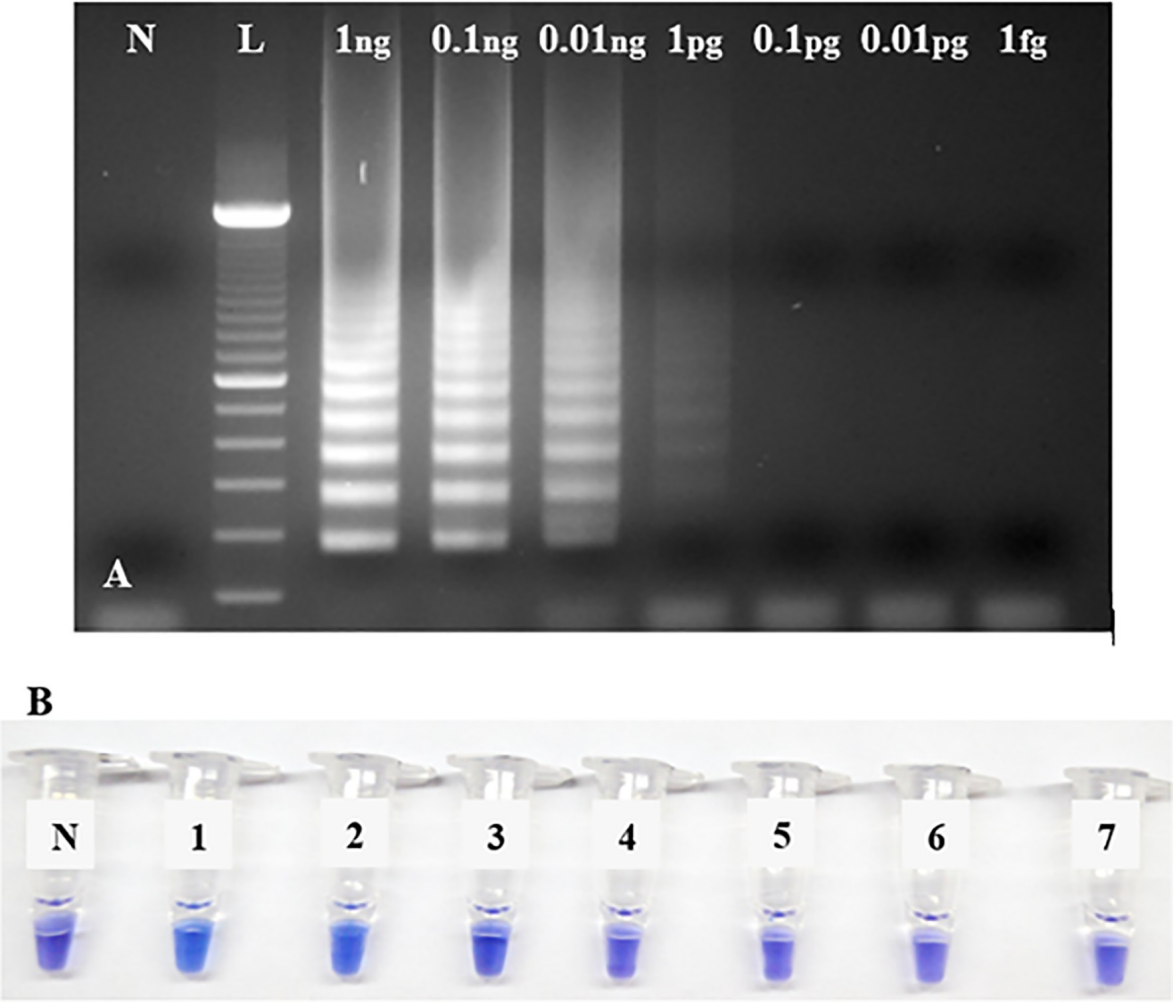
Limit of detection assay of RRV-P4 RT-LAMP performed with Bst 2.0 DNA polymerase. (A) Gel electrophoresis of RRV-P4 RT-LAMP products from RRV-P4 plasmid serially diluted from 1ng to 1fg limit. Lane N, is a non-template control (NTC, water); lane L, is a 100 bp DNA ladder. (B) Colorimetric RRV-P4 RT-LAMP using HNB. RRV-P4 plasmid was serially diluted from 1ng to 1fg. Tube N, is a non-template control (NTC, water); tube 1, 1ng/μL; tube 2, 0.1ng/μL; tube 3, 0.01ng/μL; tube 4, 1pg/μL; tube 5, 0.1pg/μL; tube 6, 0.01pg/μL; tube 7, 1fg/μL.

The LoD of RRV-P4 using Bst 2.0 DNA Polymerase is 1 pg/μL (Fig 7A) after gel electrophoresis. The colorimetric LoD using HNB was 10 pg/μL (Fig 7B). The limited HNB LoD is visible in the reaction of tube 3 (blue) compared with tube 4 (purple) and the reaction in line 4 (1pg/μL) (Fig 7).

### 3.4. LAMP specificity

**Fig 8:**
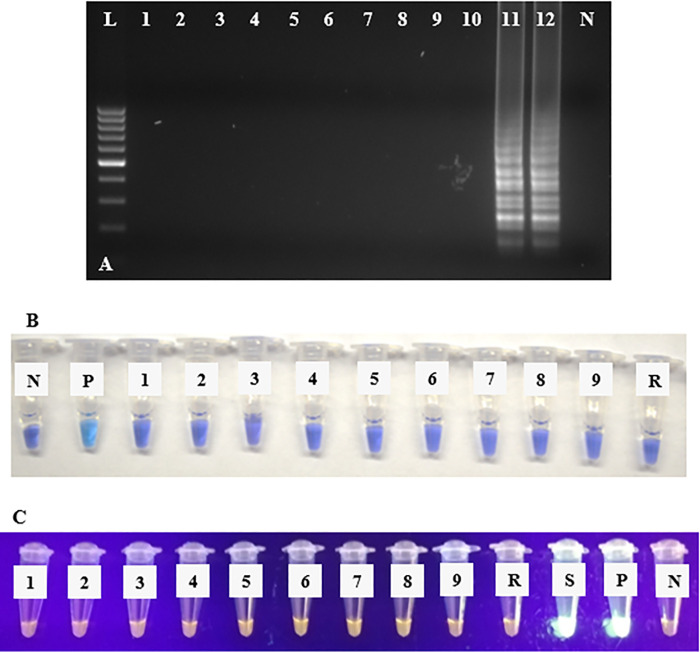
RRV-P3 RT-LAMP specificity assays using Bst 2.0 WarmStart DNA polymerase with cDNA from nine reference viruses and controls. RRV-P3 and RRV-P4 RT-LAMP results are equal. (A) Agarose gel electrophoresis of RT-LAMP products. Lane L, is a 100 bp DNA ladder, lane 1 is INSV, lane 2 is HPWMoV, lane 3 is ArMV, lane 4 is MSpV, lane 5 is TSWV, lane 6 is ApMV, lane 7, is PNRSV, lane 8 is ToRSV, lane 9 TMV (full virus names were provided in the main text for the reference viruses), lane 10 is healthy rose tissue, lane 11 is RRV symptomatic rose tissue, lane 12 is RRV-P3 plasmid, lane N is non-template control (NTC, water). (B) Colorimetric RRV-P3 RT-LAMP specificity assay using HNB using cDNA of nine reference control viruses. Tube N, is a non-template control (NTC, water), tube P is RRV-P3 plasmid, tube 1 is INSV, tube 2 is HPWMoV (formerly High plain virus), tube 3 is ArMV, tube 4 is MSpV, tube 5 is TSWV, tube 6 is ApMV, tube 7 is PNRSV, tube 8 is ToRSV, tube 9 TMV (full virus names were provided in the main text for the reference viruses), tube R, healthy rose tissue. (C) Colorimetric RRV-P3 RT-LAMP specificity assay using SYBR Green I. tube 1 is INSV, tube 2 is HPWMoV, tube 3 is ArMV, tube 4 is MSpV, tube 5 is TSWV, tube 6 is ApMV, tube 7, is PNRSV, tube 8 is ToRSV, tube 9 TMV (full virus names were provided in the main text for the reference viruses), tube R is healthy rose tissue, tube S is RRV symptomatic rose tissue, tube P is RRV-P3 plasmid, and tube N is a non-template control (NTC, water).

**Fig 9:**
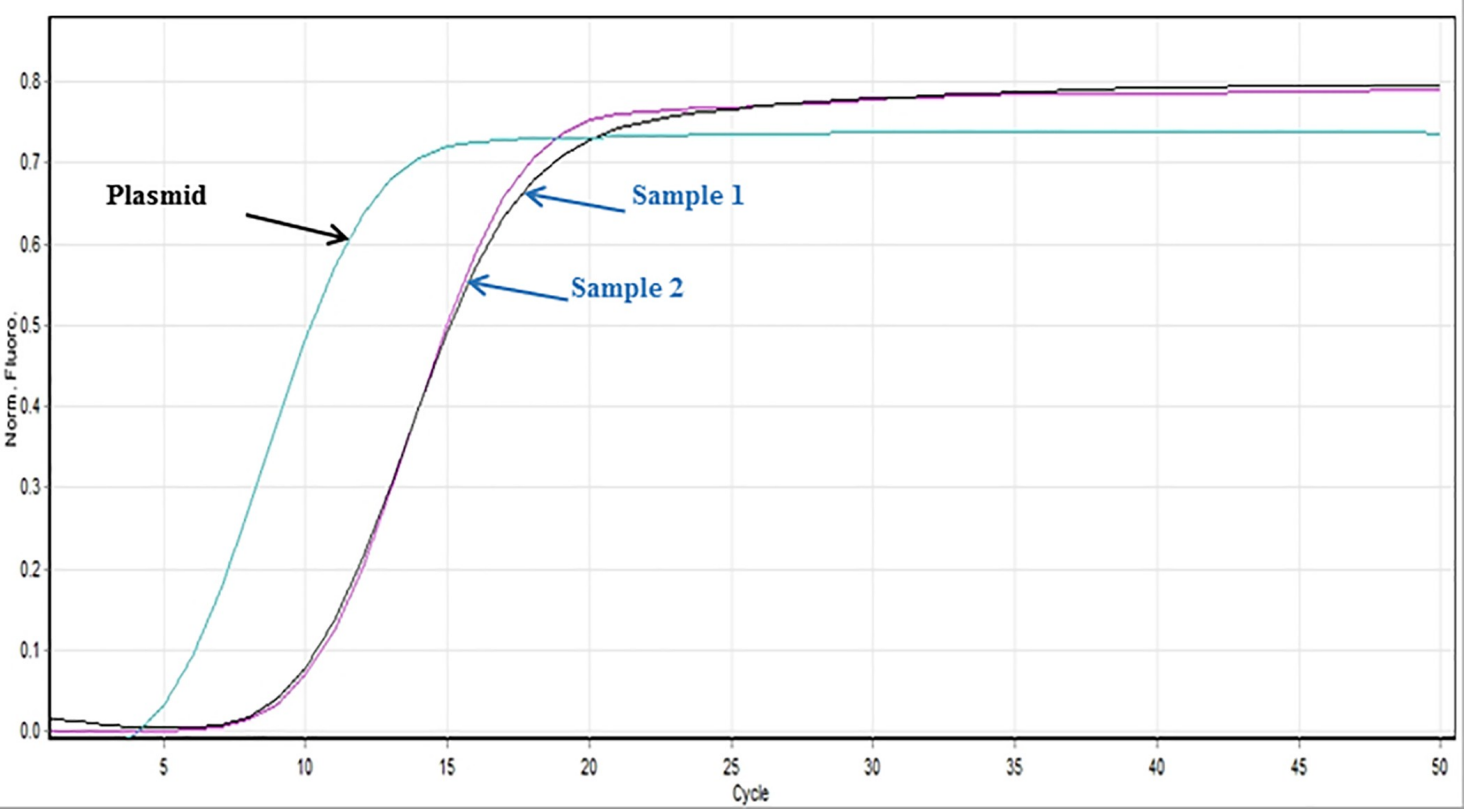
RT-qLAMP specificity assays with GspSSD2.0 DNA polymerase. (A) RT-qLAMP specificity using RNA of nine reference control viruses, healthy rose tissue, RRV symptomatic rose tissue (Sample1, 2), and RRV-P3 plasmid.

The specificity of the LAMP primers was tested using the Bst 2.0 DNA Polymerase and gel electrophoresis, HNB, and SYBR Green I. No cross-amplification was detected with reference control viruses INSV, HPWMoV, ArMV, MSpV, TSWV, ApMV, PNRSV, ToRSV, and TMV (Fig 8A, 8B, 8C). Similarly, no non-specific amplifications were detected with the total RNAs of these nine viruses using RT-qLAMP (Fig 9). The positive controls targets (1ng/μl of RRV-P3 and RRV-P4 plasmids) were amplified in the three RT-LAMP reactions (Fig 8A, 8B, 8C). The negative control cDNA from healthy rose tissue and the no template control (water) tested negative as expected.

### 3.5. Screening of infected field samples

A side-by-side comparison between RRV-P4 RT-LAMP with Bst 2.0 DNA polymerase and RT-PCR was done with 38 leaf and petal samples. The positive control (RRV-P4) and negative control (healthy rose) were included. The amplicons were visualized by gel electrophoresis and colorimetric positive reactions were not visible using HNB. Twenty-six out of 38 samples tested positive for RRV-P4 RT-LAMP with Bst 2.0 (Table 2). The complete set of 38 samples tested positive by RT-PCR. The amplification facilitators (AFs) BSA and PVP were added to the RT-LAMP reactions and allowed isothermal amplification of the expected RRV-P4 target (Table 2), however, the colorimetric HNB reaction was not visible because BSA and PVP caused an unfavorable pH change in the reaction (data not show). RNA from rose tissues was extracted using RNeasy Plant Mini Kit in this experiment. The positive control (1ng/μl of RRV-P4 plasmid) tested positive with both RRV-P4 RT-LAMP and RT-PCR while cDNA from healthy rose and the non-template control (NTC) tested negative as expected.

**Table 2:** Side-by-side comparison of RRV-P4 RT-LAMP made with Bst 2.0 DNA polymerase and RT-PCR.

N°	Rose Code	Tissue source	RT-LAMP (Bst 2.0 DNA Pol.)	RT-PCR (Dobhal et al., 2016)
**1**	R1	Leaves & petals	+	+
**2**	R2	Leaves & petals	+	+
**3**	R3	Leaves & petals	+	+
**4**	R4	Leaves & petals	+	+
**5**	R5	Leaves & petals	+	+
**6**	R6	Leaves & petals	+	+
**7**	R7	Leaves & petals	-	+
**8**	R8	Leaves & petals	-	+
**9**	R9	Leaves & petals	+	+
**10**	R10	Leaves & petals	-	+
**11**	R11	Leaves & petals	-	+
**12**	R12	Leaves & petals	-	+
**13**	R13	Leaves & petals	+	+
**14**	R14	Leaves & petals	-	+
**15**	R15	Leaves & petals	+	+
**16**	R16	Leaves & petals	+	+
**17**	R17	Leaves & petals	-	+
**18**	R18	Leaves & petals	+	+
**19**	R19	Leaves & petals	-	+
**20**	R20	Leaves & petals	-	+
**21**	R21	Leaves & petals	+	+
**22**	R22	Leaves & petals	-	+
**23**	R23	Leaves & petals	+	+
**24**	R24	Leaves & petals	+	+
**25**	R25	Leaves & petals	+	+
**26**	R26	Leaves & petals	+	+
**27**	R27	Leaves & petals	+	+
**28**	R28	Leaves & petals	+	+
**29**	R29	Leaves & petals	+	+
**30**	R30	Leaves & petals	+	+
**31**	R31	Leaves & petals	+	+
**32**	R32	Leaves & petals	+	+
**33**	R33	Leaves & petals	+	+
**34**	R34	Leaves & petals	+	+
**35**	R35	Leaves & petals	-	+
**36**	R36	Leaves & petals	+	+
**37**	R37	Leaves & petals	-	+
**38**	R38	Leaves & petals	-	+
**39**	Plasmid RRV-P4	Plasmid	+	Not tested
**40**	Healthy rose	Leaves	-	-
**41**	NTC	(water)	-	-

Results are from 38 symptomatic and no symptomatic rose samples from the Oklahoma State University RRV resistance rose varietal plot at Perkins, Oklahoma.

**Note:** (+) samples tested positive for RRV and (-) samples tested negative for RRV. NTC is non-template control.

A second side-by-side comparison tested 33 leaves, roots, and stem samples (from the Tulsa rose garden rose varietal plot, Oklahoma) with RRV-P3 RT-qLAMP using GspSSD2.0 DNA polymerase and RRV-P3 RT-LAMP with BST 2.0 DNA polymerase combined with SYBR Green I for color development post-reaction (Table 3). Twelve samples tested positive using the two methods and five samples tested positive only with RRV-P3 RT-qLAMP using GspSSD2.0. Moreover, 17 samples tested positive using RRV-P3 RT-qLAMP with GspSSD2.0, and 13 samples tested positive using RRV-P3 RT-LAMP with Bst 2.0 combined with SYBR Green I. Sixteen samples tested negative with these two methods: a) eight samples comprising leaves, bark, and roots were from two varieties (Screaming red neon and Pink surprise). The two varieties were not infected with RRV at the time of sampling. Also, ‘Hybrid 5–13’ samples 2, 4, and 5 (leaves and roots) tested negative with both of the methods but tested positive in the bark. Similarly, ‘Lemon splash’ samples 24 and 26 (stem and roots) tested negative, however, tested positive in the leaves. Another variety, Champlain (samples 19 and 20), tested negative in the leaves but positive in the bark. One out of three specimens of the variety Kiss me rose (samples 15–17) tested positive using RRV-P3 RT-qLAMP with GspSSD2.0 in leaves only (sample 15). The obtained results demonstrated the uneven distribution of RRV in rose plants. Positive controls Knock Out’ infected with RRV isolate OK1 and plasmid RRV-P3 (samples 6 and 34 respectively) tested positive with both methods. Out of the 15 rose varieties tested, 13 tested positive and two tested negative using RRV-P3 RT-qLAMP, which is the most sensitive RT-LAMP reaction in this study. No amplification or colorimetric reactions were obtained with the negative controls, healthy rose tissue, and non-template control (water) as expected.

**Table 3:** Side-by-side comparison of RRV-P3 RT-qLAMP using GspSSD2.0 DNA polymerase and colorimetric RRV-P3 RT-LAMP using Bst 2.0 DNA polymerase with SYBR Green I.

N°	Rose variety	Tissue source	RT-qLAMP (GspSSD2.0)	RT-LAMP (Bst 2.0+SYBR Green I)
**1**	Caroline Hunt (1)	Leaves	+	-
**2**	5–13 hybrid (1)	Leaves	-	-
**3**	5–13 hybrid (2)	Stem	+	+
**4**	5–13 hybrid (2)	Leaves	-	-
**5**	5–13 hybrid (2)	Roots	-	-
**6**	Knock out OK1-IHop (1)	Leaves	+	+
**7**	Screaming red neon (1)	Leaves	-	-
**8**	Screaming red neon (2)	Roots	-	-
**9**	Screaming red neon (2)	Stem	-	-
**10**	Screaming red neon (2)	Leaves	-	-
**11**	Pink surprise (1)	Leaves	-	-
**12**	Pink surprise (2)	Leaves	-	-
**13**	Pink surprise (2)	Stem	-	-
**14**	Pink surprise (2)	Roots	-	-
**15**	Kiss me rose (1)	Leaves	+	-
**16**	Kiss me rose (2)	Stem	-	-
**17**	Kiss me rose (2)	Roots	-	-
**18**	Dulchen (1)	Leaves	+	+
**19**	Champlain (1)	Leaves	-	-
**20**	Champlain (2)	Stem	+	+
**21**	I06-20-14-3x PH (1)	Leaves	+	+
**22**	I06-20-14-3x PH (1)	Roots	+	+
**23**	Lemon splash (1)	Leaves	+	+
**24**	Lemon splash (2)	Stem	-	-
**25**	Lemon splash (2)	Leaves	+	+
**26**	Lemon splash (2)	Roots	-	-
**27**	Above & Beyond (1)	Leaves	+	-
**28**	Above & Beyond (2)	Roots	+	+
**29**	Above & Beyond (2)	Stem	+	+
**30**	Como Park (1)	Leaves	+	-
**31**	5–21 hybrid (1)	Leaves	+	-
**32**	Pink double knockout (1)	Leaves	+	+
**33**	Apricot drift (1)	Leaves	+	+
**34**	Plasmid RRVP-3	Plasmid	+	+
**35**	Healthy rose	Leaves	-	-
**36**	NTC	(water)	-	-

Results are from 33 rose samples from the Tulsa rose garden rose varietal plot, Oklahoma.

**Note:** (+) samples tested positive for RRV, (-) samples tested negative for RRV, (1) first sampling (2) second sampling. NTC is non-template control.

A third side-by side-comparison contrasts RRV-P3 RT**-**qLAMP with GspSSD2.0, RRV-P3 RT**-**LAMP with Bst 2.0 with SYBR Green I, and RT-PCR (Table 4). Varieties Dulchen (sample 7), Pink double knockout (11), Apricot drift (sample12), Lemon splash (sample 14), Above & beyond (samples15, 16) tested positive to the three methods. Variety Kiss me Rose and Hybrid 5–21 (samples 6, 10) tested positive only to RRV-P3 RT**-**qLAMP with GspSSD2.0 and RT-PCR. Varieties Caroline Hunt and Como Park tested positive to RRV-P3 RT**-**qLAMP with GspSSD2.0 only. Positive controls plasmid RRV-P3 tested positive with the three methods. The negative controls did no amplify or generated a colorimetric reaction with healthy rose tissue, and the NTC (water) as expected. In general, detection results using RRV-P3 RT**-**qLAMP with GspSSD2.0 and RT-PCR agreed with the LoD reported and the varietal results were consistent across Tables 3 and 4. The method for direct antigen-capture was consistently trapping RRV directly in plastic from different tissue sources of roses. The addition of SYBR Green I post RT-LAMP reaction generated color change toward fluorescent green in positive amplification, while no change of color or steady-orange was consistent in all negative reactions (no amplification), healthy rose. Amplification and colorimetric reaction (SYBR Green I) were obtained with the RVV-P3 plasmid.

**Table 4:** Side-by-side comparison of RRV-P3 RT-qLAMP using GspSSD2.0, colorimetric RRV-P3 RT-LAMP using Bst 2.0, and RT-PCR.

N°	Rose variety	Tissue source	RT-qLAMP (GspSSD2.0)	RT-LAMP (Bst+SYBR Green I)	RT-PCR (Dobhal et al., 2016)
**1**	Caroline Hunt	Leaves	+	-	-
**2**	5–13 hybrid	Leaves	-	-	-
**4**	Screaming neon red	Leaves	-	-	-
**5**	Pink surprise	Leaves	-	-	-
**6**	Kiss me rose	Leaves	+	-	+
**7**	Dulchen	Leaves	+	+	+
**8**	Champlain	Leaves	-	-	-
**9**	Como park	Leaves	+	-	-
**10**	5–21 hybrid	Leaves	+	-	+
**11**	Pink double knockout	Leaves	+	+	+
**12**	Apricot dnift	Leaves	+	+	+
**13**	Lemon splash	Stem	-	-	
**14**	Lemon splash	Leaves	+	+	+
**15**	Above & beyond	Roots	+	+	+
**16**	Above & beyond	Stem	+	+	+
**17**	Screaming red neon	Leaves	-	-	-
**18**	Healthy rose	Leaves	-	-	-
**19**	Plasmid RRV-P3	Plasmid	+	+	+
**20**	NTC	(water)	-	-	-

Results are from seventeen field samples of roses from the Tulsa rose garden rose varietal plot, Oklahoma.

NTC is non-template control.

## 4. Discussion

This study explores, describes, and demonstrates the development of specific, sensitive, and relatively easy-to-use RRV RT-LAMPs. The study used two sets of primers for RT-LAMP amplification of RRV-P3 and RRV-P4 genes, which were tested with Bst 2.0 and GspSSD2.0 DNA polymerases. These methods have the potential to be field deployable. RT-LAMP was also tested using SYBR green I and HNB to generate qualitative colorimetric reactions, and an RT-qLAMP was tested.

The viral nucleic acids, in this case, RNA, required for RT-LAMP reactions is commonly extracted from plant tissue using commercially available kits. However, RNA isolated from roses may carry phenolic compounds, starch, other carbohydrates, pigments, and other plant residues in high levels. These compounds inhibit the targeted cDNA and/or DNA amplification because they interact with the viral nucleic acids and the reaction mix proteins causing oxidation and degradation of the RNA [22]. All of which decreases the quality of the samples and causes inconsistent results and concerns about whether all RRV isolates are detectable. Dobhal et al. [23], enhanced RRV RT-PCR detection by adding DNA AFs such as BSA and PVP minimizing the inhibitory effect of putative rose components. However, it was observed the colorimetric RT-LAMP- HNB reaction did not develop a visually distinguishable (sharp) change of color between positive and negative reactions when BSA and PVP were added to the reaction, yet obtained amplification is detectable by gel electrophoresis (data not shown). The development of color in colorimetric RT-LAMP- HNB reactions relies on a change in pH occurring during the reaction, and the incorporation of AFs such as BSA and PVP may be interfering with the acidification of the reaction [24].

The direct antigen-capture method reported by Babu et al. [18] allowed rapid and consistent trapping of RRV virions directly on plastic (inner PCR tube surface). The method washes the rose tissue impurities and improves the quality of RNA used in RT-LAMP reactions and it substantially reduces the sample processing time. The method also reduces viral RNA extraction time for LAMP, RPA, and/or RT-PCR to less than 5 min. Simple RNA extraction methods combined with RT-LAMP have been performed to reduce the detection time. The wooden toothpick was tested to collect Fig mosaic virus (FMV) for direct transfer to RT-LAMP; this method can detect the virus in 30 min; however, sampling should be taken from a symptomatic plant region; otherwise, detection could be a negative result [25]. In contrast, the direct antigen capture method uses a mix of plant samples including asymptomatic samples that can still be detected by RT-LAMP. In summary, the direct antigen-capture method applied to RT-LAMP allowed the RNA reverse transcription and amplification of targeted DNA with no need for BSA and PVP enhancers. Furthermore, the direct antigen-capture did not interfere with pre-reaction pH-sensitive dye (hydroxy naphthol blue) and post-reaction dye (SYBR Green I) in colorimetric RT-LAMP reactions.

The LAMP primers were design based on RNA3 and RNA4 of the RRV genome because these segments were highly conserved among the isolates available in the NCBI database. In addition, comparing RNA1 (RNA dependent RNA polymerase -RdRp) and RNA3 (nucleocapsid), RNA3 could be encoding high levels of nucleocapsid protein; therefore, the RNA3 segment is a good candidate for targeting the RRV [7, 26]. The use of only four primers per gene-kit facilitated RT-LAMP optimization, assay design, and application of optimized RT-LAMP parameters i.e. optimal outer and inner primer ratios, reaction temperature, and best concentration of magnesium sulfate, which successfully favored the amplification of the expected RRV-P3 and RRV-P4 targets at 64°C and 66.5°C respectively (Figs 2, 3 and 4). Having RT-LAMP performing in a broad range of reaction temperatures reduces errors due to temperature variability that might occur associated with equipment calibration. The selected MgSO4 concentration (4mM) allowed steady amplification of both RRV-P3 and RRV-P4 targets. The optimal concentration of the inner and outer LAMP primers is 0.8μM and 0.2 μM respectively, equivalent to a 4:1 primer ratio. The described Bst 2.0 driven RT-LAMP amplification can be translated to the field or nursery testing if using the fluorescent (SYBR green I). The pH-sensitive dyes (HNB) are less recommended. An advantage of LAMP colorimetric results is that can be judged either at daylight or UV light. A critical difference to consider if pursuing field-testing is that SYBR Green I has to be added at the end of the RT-LAMP reaction, while HNB must be added to the mix before the RT-LAMP reaction starts (Fig 1). To mitigate the risk of contamination while opening the tubes a top layer of mineral oil was added to all reactions. SYBR green I allowed clear visual discrimination between infected and healthy samples compared to HNB (Figs 5B, 6B and 6C). The visual LoD of SYBR green I and HNB are 0.01ng/μL and 0.1pg/Ll, respectively.

The LoD between the two LAMP chemistries studied was different. Bst 2.0-based RT-LAMP detected RRV to 1pg/μL while GspSSD2.0 RT-qLAMP allows detection to1fg/μL. The RT-qLAMP assay from this study and the RRV RT-PCR reported by Dobhal et al. [11] have equivalent LoDs (1fg/μL) (Fig 8).

The two RRV-RT-LAMP sets of primers did not cross-react with eight reference control virus commonly co-infecting rose viruses, one taxonomically related emaravirus to RRV, and healthy rose tissue, which confirmed the predicted specificity pairwise analysis results obtained *in-silico* using BLASTn. In terms of specificity, no difference between LAMP primer sets RRV-P3 gene (viral coat protein) and RRV-P4 gene (movement protein) was detected (Fig 9).

The RT-qLAMP assay is relatively less time-consuming because was combined with the direct antigen-capture or direct trapping in plastic which takes 10–15 minutes for a batch of 1–15 samples. The one-step GspSSD2.0 RT-qLAMP reaction takes approximately 1 hour and uses RNA directly as a template while the two-step RT-PCR reaction time takes circa 2 hours and uses cDNA as a template. cDNA requires an additional 45 min to 1 hour. In this research, GspSSD2.0 RT-qLAMP was performed with a thermocycler (RotorGene 6000), but fluorescence recordings can be also performed using a simpler fluorescent reader that allows on-site testing of doubtful symptomatic rose plants. The side-by-side comparison of RT-LAMP and RT-PCR [11] showed inconsistent detection of RRV by RRV-P4 RT-LAMP with Bst 2.0- polymerase if compared to RT-PCR (Table 2). Twelve samples tested negative out of 38 RT-PCR positives. The discrepancy between the two methods (13 samples) is due to their differences in LoD, 1pg/μl for RT-LAMP, and 1fg/μl for RT-PCR [11]. Improvement in LAMP primer design and adding loop primers (LF and LB) would improve the LoD of the method. A second side-by-side comparison between RRV-P3 RT-qLAMP using GspSSD2.0 and the colorimetric RRV-P3 RT-LAMP using Bst 2.0 combined with SYBR Green was performed with 33 rose samples (Table 3). Twelve samples tested positive using the two methods and five samples tested positive only with RRV-P3 RT-qLAMP GspSSD2.0. This discrepancy is due to the difference in LoD between RT-qLAMP (1fg/μl) and RT-LAMP using BST 2.0 with SYBR Green I (0.1pg/μl), Figs 5 and 6. Another group of 17 samples tested positive using RRV-P3 RT-qLAMP with GspSSD2.0, and thirteen samples tested positive using RRV-P3 RT-LAMP with Bst 2.0 combined with SYBR Green I, other 16 samples tested negative with these two methods. Testing leaves, bark, and roots from different varieties demonstrated the uneven distribution of RRV in rose plants which is detailed in Table 3. Regarding the uneven distribution of the virus, sampling should be performed from different plant parts to have a higher possibility to detect RRV. However, further research is needed to determine the best sampling procedure for RRV detection.

A third side-by-side comparison of RRV-P3 RT-qLAMP GspSSD2.0, colorimetric RRV-P3 RT-LAMP Bst 2.0 with SYBR Green I, and RT-PCR was made targeting seventeen rose samples collected at the RRV resistance rose varietal plot located at the Tulsa rose garden (Table 4). This experiment also shows discrepancies in detection among the three methods. The GspSSD2.0 detected 2 RRV positives out of the 17 samples that were not detected by the colorimetric Bst 2.0 DNA Polymerase plus SYBR Green I and RT-PCR. These results are consistent with the low LoD of Bst 2.0 reported and the varietal results were consistent across Tables 3 and 4.

The uneven distribution of RRV in plants may be the cause of RRV passing not adverted by RT-PCR. In general, RRV was detected from leaves, petals, stems (bark), and roots. Consistent amplification of RRV was obtained from leaves, however, inconsistencies using RT-PCR point toward the uneven distribution of RRV in their hosts and the need for a statistically based sampling method.

In general, the obtained RT-LAMP results agree with developed LAMPs reported for plant viruses such as banana bunchy top virus (BBTV), banana streak viruses (BSVs), cucumber mosaic virus (CMV), tomato chlorosis virus (ToCV), potato virus Y (PVY), tobacco etch virus (TEV), tobacco mosaic virus (TMV), and rice ragged stunt virus (RRSV). These LAMPs used the Bst 2.0 DNA polymerase [27–29]. Another polymerase, GspSSD2.0 was also reported for LAMPs assays for turnip yellows virus (TuYV), chrysanthemum stem necrosis virus (CSNV), fig mosaic virus (FMV), little cherry virus (LChV), and sugarcane mosaic virus (SCMV) [25, 30–34].

## 5. Conclusion

This study explored RT-LAMP with two DNA polymerases, RT-qLAMP with GspSSD2.0 which can be confirmed by gel electrophoresis, and colorimetric RT-LAMP with Bst 2.0. Four different dyes were tested, but visible colorimetric reactions were obtained only when RT-LAMP Bst 2.0 was combined with SYBR I or HNB. The last is less recommended since the change of color is low contrast. RT-qLAMP with GspSSD2.0 offers LoD equal to RT-PCR, takes a shorter time since it works with RNA directly and one-step reaction, however, does not support colorimetry. RT-LAMP with Bst 2.0. does not have an LoD as low as RT-PCR, but it supports colorimetry. Colorimetric RT-LAMP Bst2.0 also takes additional reaction time since requires a reverse-transcription step. In general, the tested isothermal LAMPs have the potential for field application, monitoring virus-free germplasm in nurseries, selection of RRV-resistant germplasm, biosecurity surveillance at points of entry, and microbial forensics.

## Supporting information

S1 Table Primer explorer selection parameters for LAMP primer set. (DOCX)

S1 Raw images (PDF)
