# Supplementary material for: Probing Loop-Mediated Isothermal Amplification (LAMP) targeting two gene-fragments of rose rosette virus
Source: PLoS One. 2021 Nov 29;16(11):e0256510. doi: 10.1371/journal.pone.0256510 (PMC8629277; doi:10.1371/journal.pone.0256510)
Supplement: S1 Table — (DOCX) [file pone.0256510.s001.docx]

| Selection Parameters | F1 – B1 | F2 – B2 / F3 – B3 | Loop Primer |
| --- | --- | --- | --- |
| Temperature (Tm) | 65°C | 60°C | 65°C |
| Delta G (ΔG) | 64 – 66°C | 59 – 61°C | 64 – 66°C |
| CG % | Less than -4 kcal/mol  Optimal 50 – 60% | | |
| Secondary structure | Should not be complementary in 3 ' | | |
| Distance between primers | F1 – F2  40 – 60bp | F1 – F2  120bp | F1 – F2  0 – 60bp |

**S1 Table. Primer explorer selection parameters for LAMP primer set.**
