# Supplementary material for: Probing Loop-Mediated Isothermal Amplification (LAMP) targeting two gene-fragments of rose rosette virus
Source: PLoS One. 2021 Nov 29;16(11):e0256510. doi: 10.1371/journal.pone.0256510 (PMC8629277; doi:10.1371/journal.pone.0256510)
Supplement: S1 Raw images — (PDF) [file pone.0256510.s002.pdf]

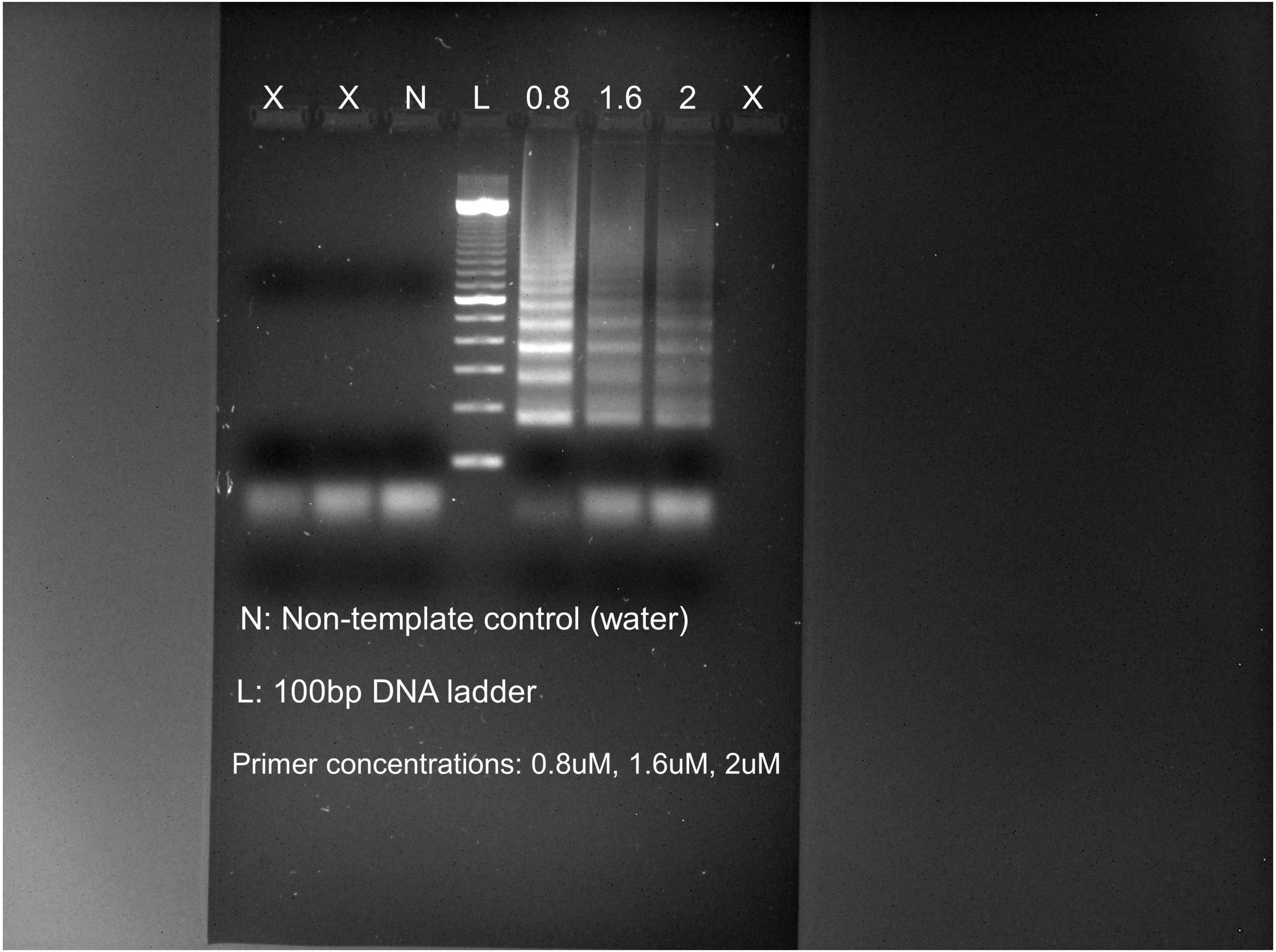
X X N L 0.8 1.6 2 X
N: Non-template control (water)
L: 100bp DNA ladder
Primer concentrations: 0.8uM, 1.6uM, 2uM

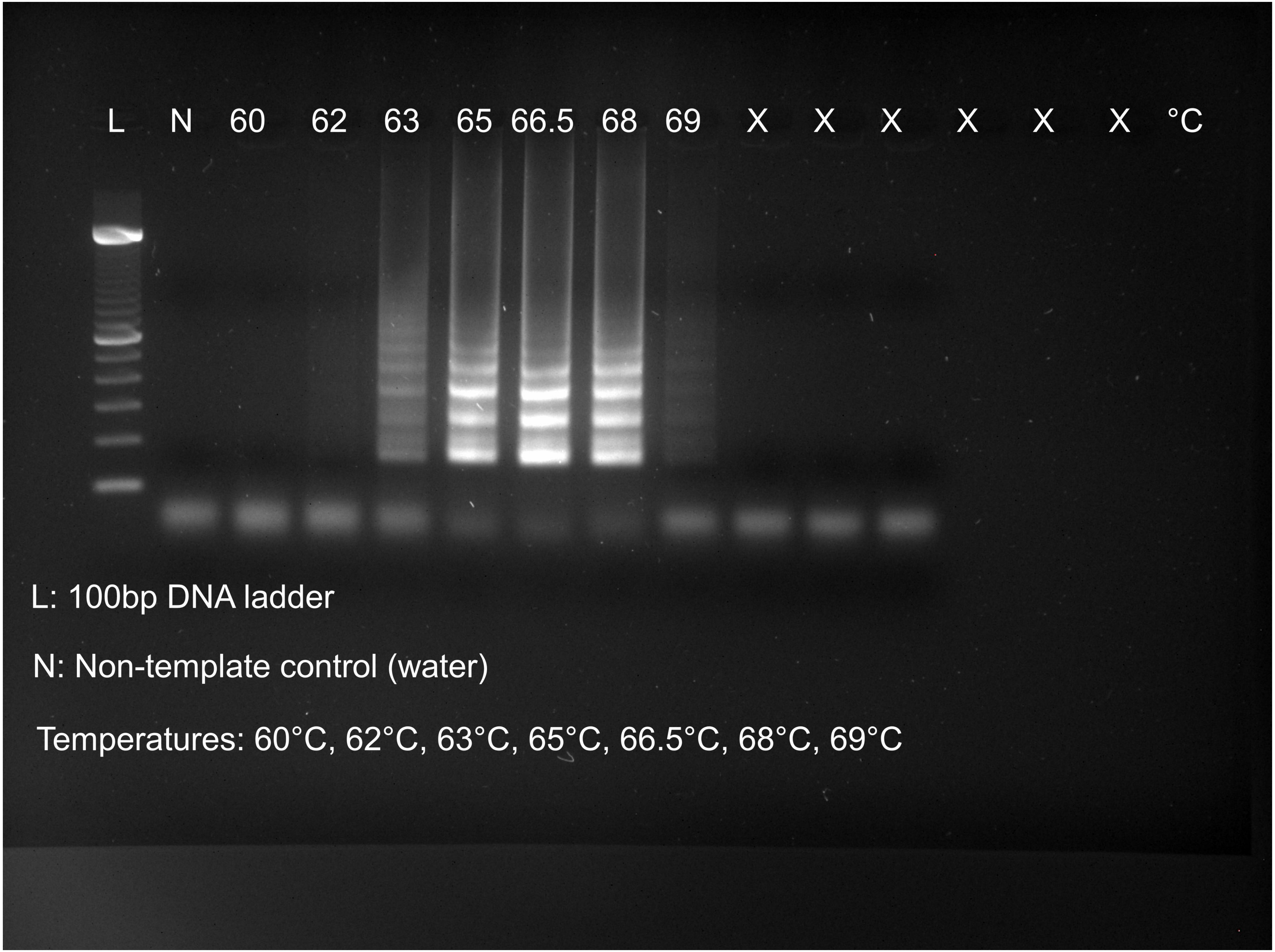
L N 60 62 63 65 66.5 68 69 X X X X X X °C
L: 100bp DNA ladder
N: Non-template control (water)
Temperatures: 60°C, 62°C, 63°C, 65°C, 66.5°C, 68°C, 69°C

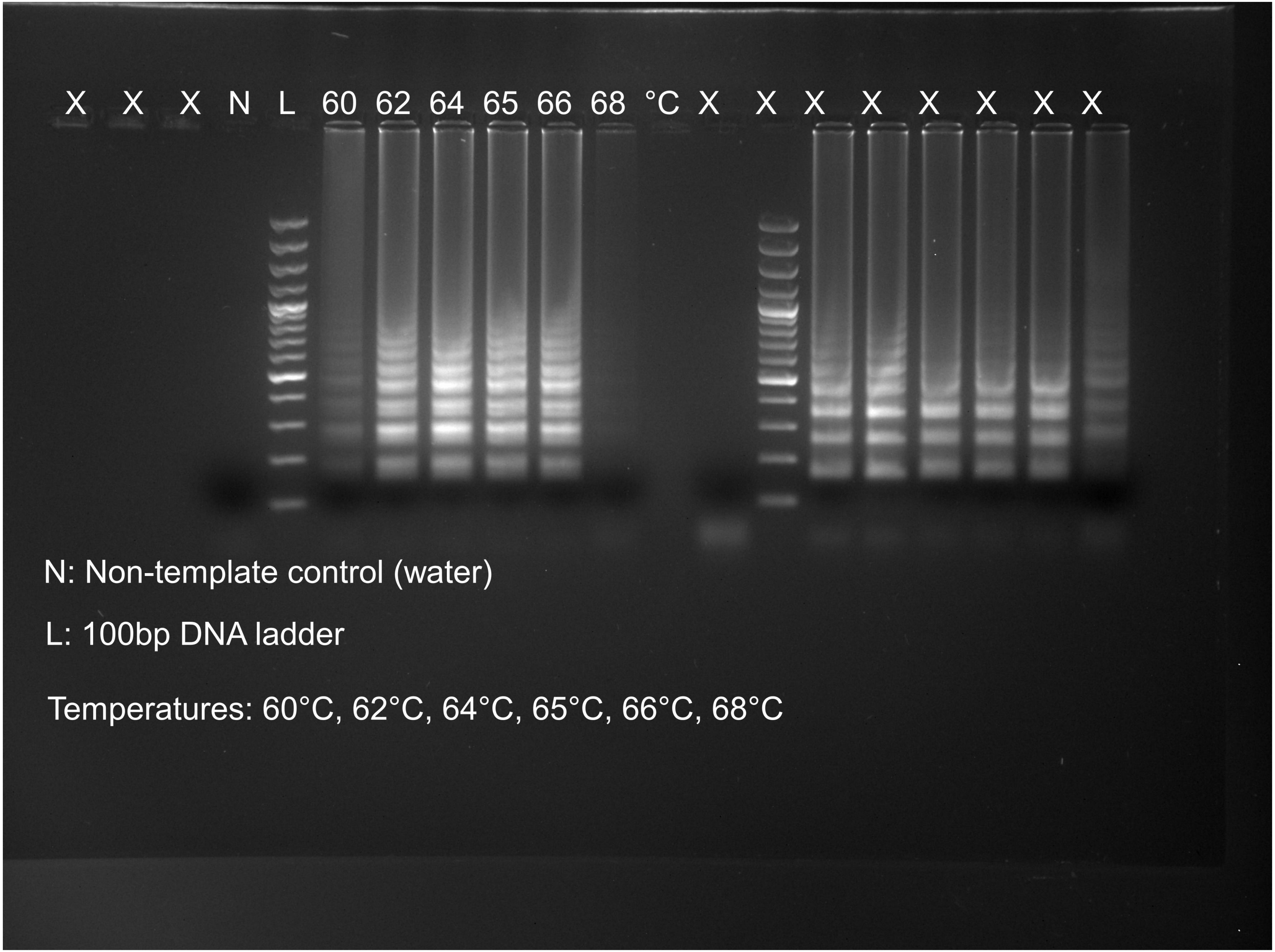
X X X N L 60 62 64 65 66 68 °C X X X X X X X X
N: Non-template control (water)
L: 100bp DNA ladder
Temperatures: 60°C, 62°C, 64°C, 65°C, 66°C, 68°C

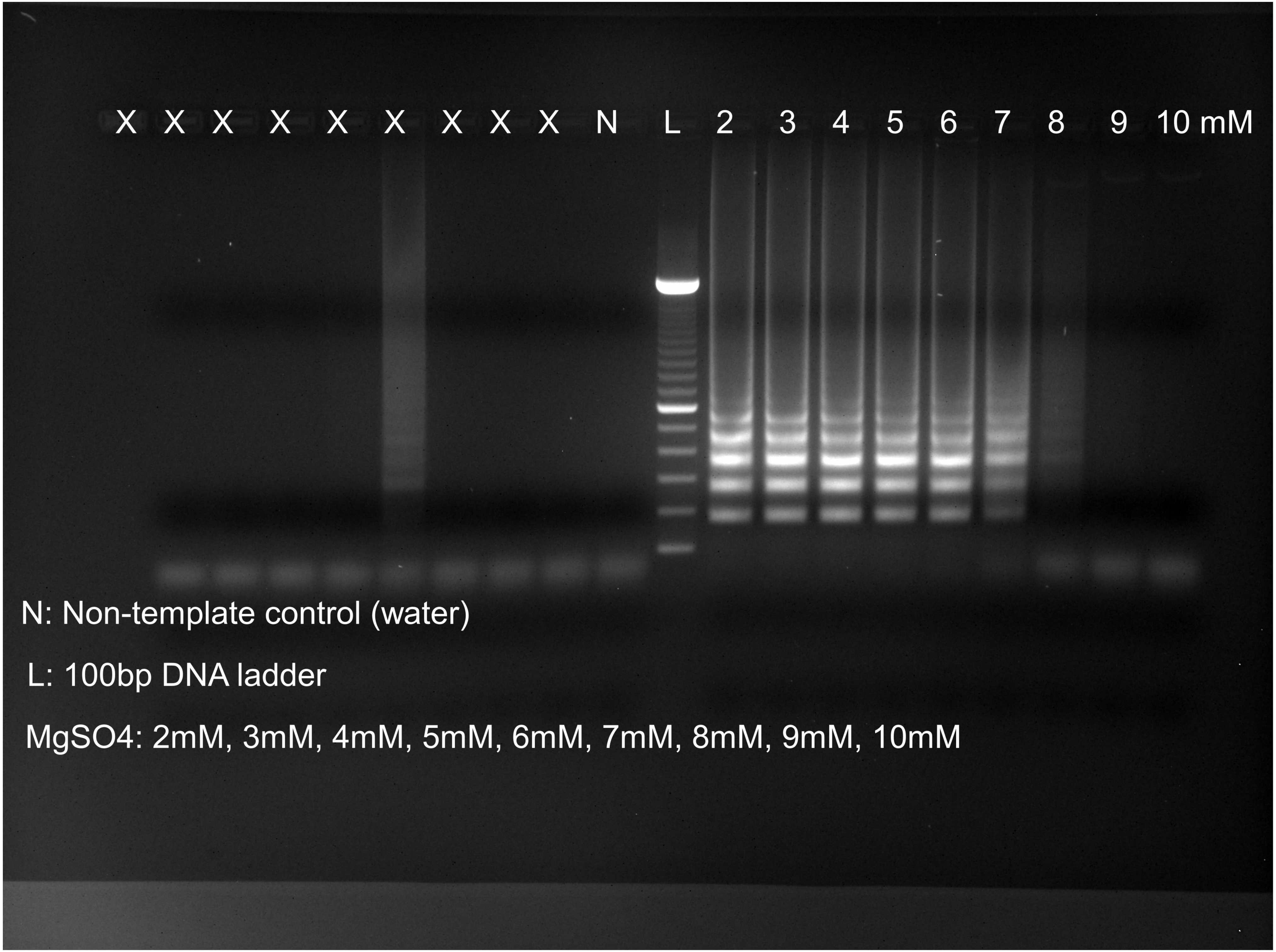
X X X X X X X X X N L 2 3 4 5 6 7 8 9 10 mM
N: Non-template control (water)
L: 100bp DNA ladder
MgSO4: 2mM, 3mM, 4mM, 5mM, 6mM, 7mM, 8mM, 9mM, 10mM

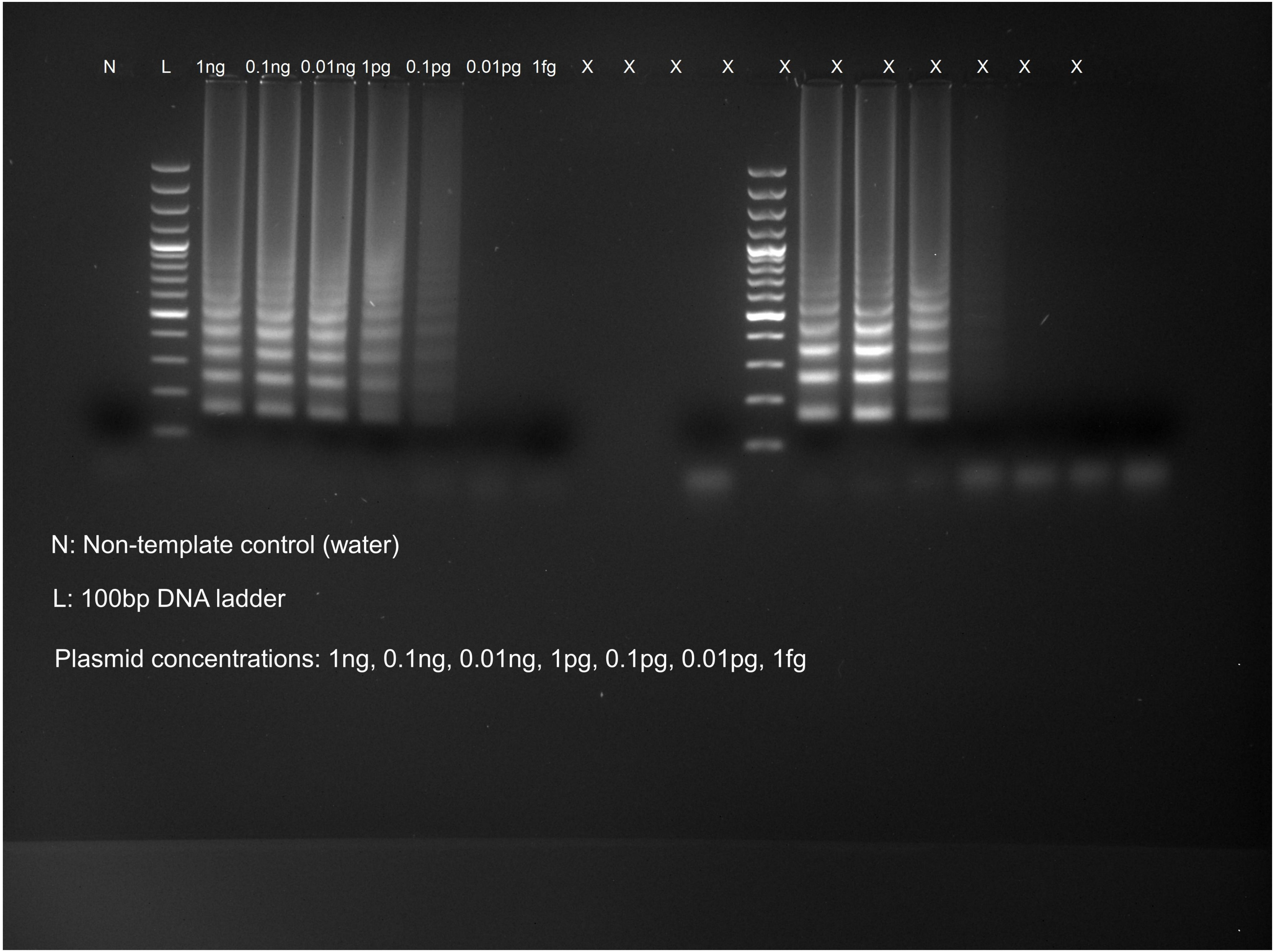

N: Non-template control (water)

L: 100bp DNA ladder

Plasmid concentrations: 1ng, 0.1ng, 0.01ng, 1pg, 0.1pg, 0.01pg, 1fg

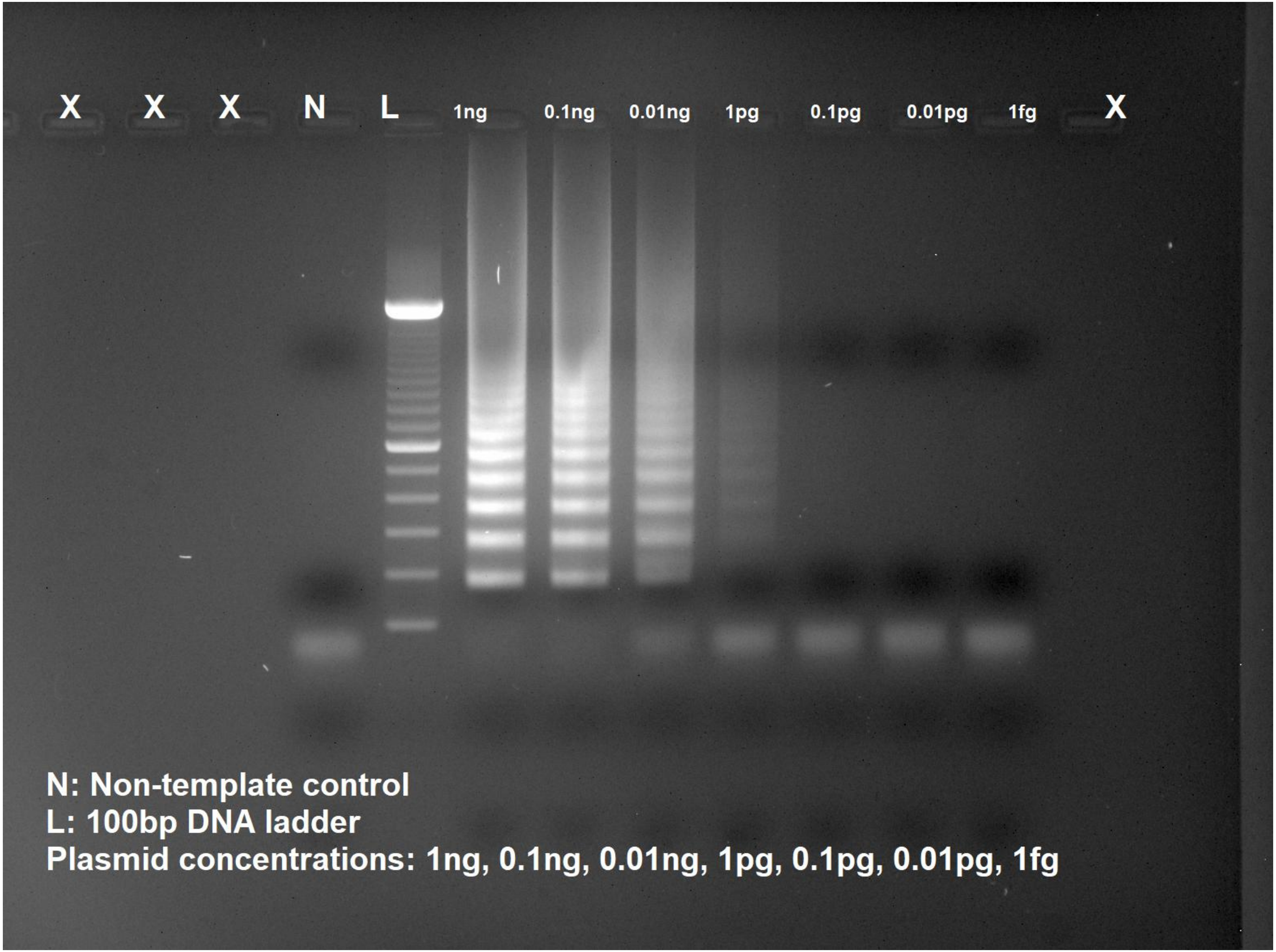
X X X N L 1ng 0.1ng 0.01ng 1pg 0.1pg 0.01pg 1fg X
N: Non-template control
L: 100bp DNA ladder
Plasmid concentrations: 1ng, 0.1ng, 0.01ng, 1pg, 0.1pg, 0.01pg, 1fg

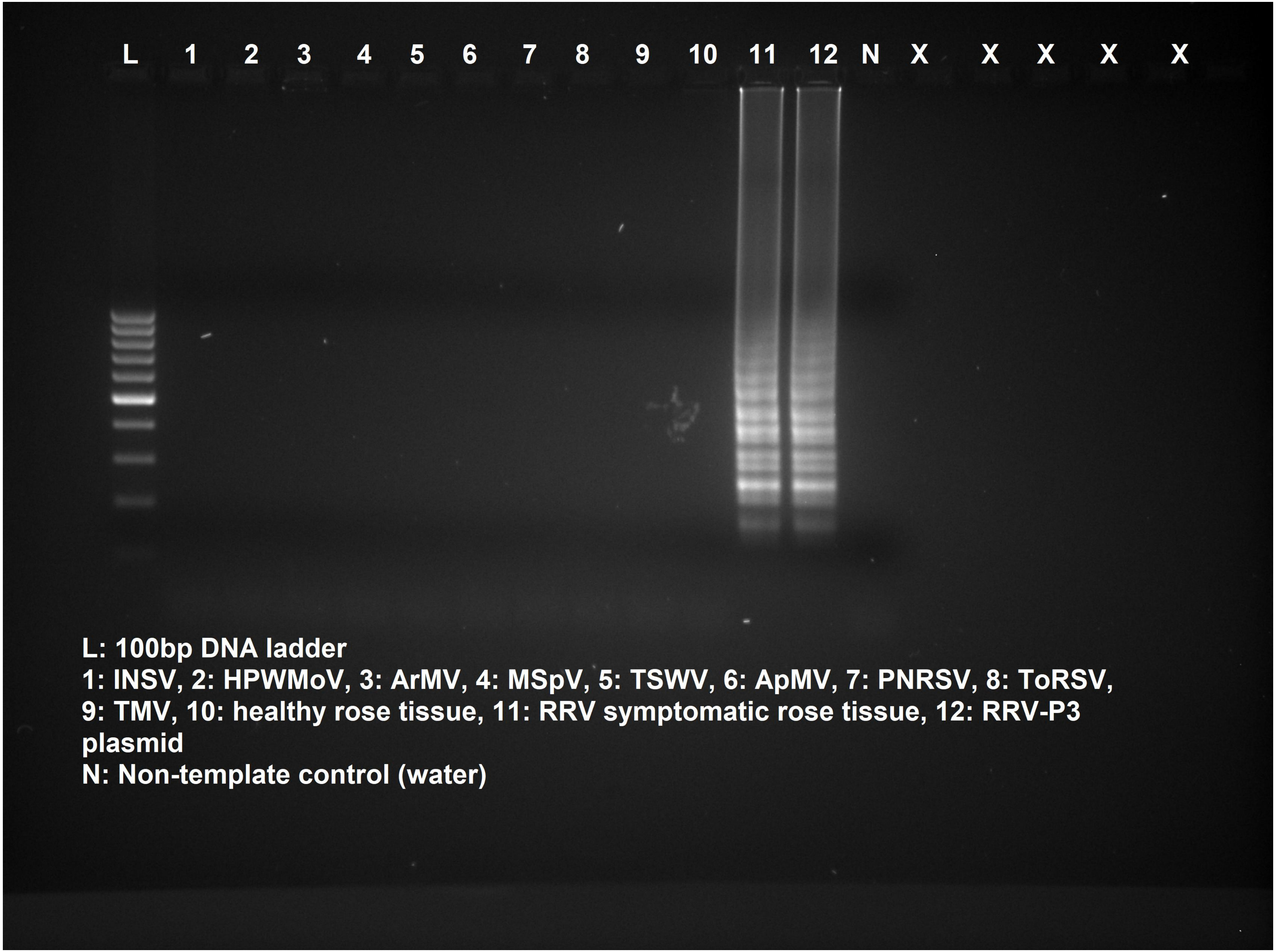

L: 100bp DNA ladder
1: INSV, 2: HPWMoV, 3: ArMV, 4: MSpV, 5: TSWV, 6: ApMV, 7: PNRSV, 8: ToRSV, 9: TMV, 10: healthy rose tissue, 11: RRV symptomatic rose tissue, 12: RRV-P3 plasmid
N: Non-template control (water)
